# Multi-dimensional task recognition for human-robot teaming: literature review

**DOI:** 10.3389/frobt.2023.1123374

**Published:** 2023-08-07

**Authors:** Prakash Baskaran, Julie A. Adams

**Affiliations:** Collaborative Robotics and Intelligent Systems Institute, Oregon State University, Corvallis, OR, United States

**Keywords:** human-robot teaming, task recognition, activity recognition, wearable sensors, machine learning

## Abstract

Human-robot teams collaborating to achieve tasks under various conditions, especially in unstructured, dynamic environments will require robots to adapt autonomously to a human teammate’s state. An important element of such adaptation is the robot’s ability to infer the human teammate’s tasks. Environmentally embedded sensors (e.g., motion capture and cameras) are infeasible in such environments for task recognition, but wearable sensors are a viable task recognition alternative. Human-robot teams will perform a wide variety of composite and atomic tasks, involving multiple activity components (i.e., gross motor, fine-grained motor, tactile, visual, cognitive, speech and auditory) that may occur concurrently. A robot’s ability to recognize the human’s composite, concurrent tasks is a key requirement for realizing successful teaming. Over a hundred task recognition algorithms across multiple activity components are evaluated based on six criteria: sensitivity, suitability, generalizability, composite factor, concurrency and anomaly awareness. The majority of the reviewed task recognition algorithms are not viable for human-robot teams in unstructured, dynamic environments, as they only detect tasks from a subset of activity components, incorporate non-wearable sensors, and rarely detect composite, concurrent tasks across multiple activity components.

## 1 Introduction

Human-robot teams (HRTs) are often required to operate in dynamic, unstructured environments, which poses a unique set of task recognition challenges that must be overcome to enable a successful collaboration. Consider a post-tornado disaster response that requires locating, triaging and transporting victims, securing infrastructure, clearing debris, and locating and securing potentially dangerous goods from looting (e.g., weapons at a firearms shop, drugs at a pharmacy). Achieving effective human-robot collaboration in such scenarios requires natural teaming between the human first responders and their robot teammates (e.g., quadrotors or ground vehicles). An important element of such teaming is the robot teammates’ ability to infer the tasks performed by the human teammates in order to adapt their interactions autonomously based on their human teammates’ state.

Tasks performed by human teammates can be classified into two categories: 1) Atomic, and 2) Composite. *Atomic tasks* are simple activities that are either short in duration, or involve repetitive actions that cannot be decomposed further. A *Composite task* aggregates multiple atomic actions or activities into a more complex task (Chen K. et al., 2021). For example, *Clearing a dangerous item* is a composite task comprised of several atomic tasks: a) Scanning the environment for suspicious items, b) Evaluating the threat level, c) Taking a picture, d) Discussing with the incident commander via walkie-talkie, and e) moving on to the next area.

**FIGURE 1:**
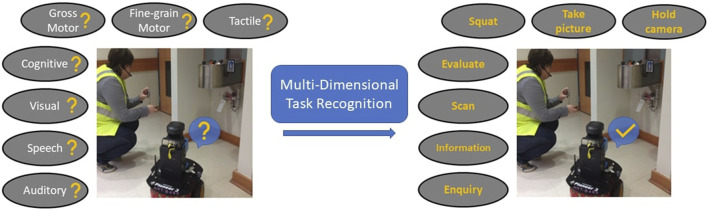
An example of a multi-dimensional task recognition framework enabling a robot to detect a human teammate’s tasks across all activity components when the HRT is clearing a dangerous item.

Humans conduct tasks using a breadth of their capabilities, as depicted in Figure 1. Depending on the complexity, the tasks can involve multiple activity components: gross motor, fine-grained motor, tactile, cognitive, visual, speech, and auditory (Heard et al., 2018a). For example, the *Clearing a dangerous item* task aggregates a *visual* component of scanning the environment to locate the item, a *cognitive* component of evaluating the item’s threat level, taking a picture task involves *gross motor*, *fine-grained motor* and *tactile* components, while discussing the next steps with the incident commander via walkie-talkie involves *auditory* and *speech* components for listening to queries and providing information.

HRTs often perform a wide variety of tasks, such that the set of tasks performed by human teammates may involve differing combinations of multi-dimensional activity components. The focus on disaster response environments, which are dynamic, uncertain, and unstructured, does not permit the use of environmentally embedded sensors (e.g., motion capture systems and cameras). Wearable sensors are a viable alternative that facilitate gathering the objective data necessary to identify a human’s task. Robots will require a multi-dimensional task recognition algorithm capable of detecting composite tasks composed of differing combinations of the activity components. Current state-of-the-art HRT task recognition using wearable sensors generally focuses on gross motor and some fine-grained atomic tasks. Prior research identified visual, cognitive, and some auditory tasks using wearable sensors; however, none of those methods recognize composite tasks across all activity components.

Humans often complete multiple tasks concurrently (Chen K. et al., 2021). For example, while *clearing dangerous items*, a human teammate may receive a *communication request* from the incident command center seeking important information and the teammate will be required to do both tasks simultaneously. Detecting this task concurrency will allow robots to better adapt to their teammate’s interactions, priorities or appropriations, which will improve the team’s overall collaboration and performance. A limitation of most existing algorithms assume that the human performs a single composite task at a time.

Individual differences are expected, even if the humans receive identical training, resulting in differing task completion steps, such as different atomic tasks being completed in different orders or with differing completion times. These differences can result in one task being mapped to multiple different sensor readings. For example, while *applying tourniquet*, a first responder may skip securing the excess band, as it is not a necessary step to stop the bleeding, while another responder may pack the wound with gauze, an additional step. Algorithmically identifying such individual differences is challenging. The existing approaches to addressing individual differences only considered trivial gross-motor and fine-grained motor atomic tasks (Mannini and Intille, 2018; Akbari and Jafari, 2020; Ferrari et al., 2020), not composite tasks involving multiple activity components.

Human responders train to respond to disasters, but each disaster differs and often requires the completion of unique tasks or tasks in a different manner. Performing tasks for which the robot has not been previously trained are called *out-of-class tasks* (Laput and Harrison, 2019). Misclassification of an out-of-class task can result in a robot adapting its behavior incorrectly, causing more harm than good.

This literature review evaluates task recognition algorithms within the context of HRTs, operating in uncertain, dynamic environments, by developing a set of relevant criteria. Sections 2, 3 provide the necessary background on task recognition and discuss prior literature reviews, respectively. Section 4 provides an overview of the relevant task recognition metrics, while Section 5 reviews relevant algorithms. Section 6 discusses the limitations of the current approaches, while Sections 7, 8 provide insights for future directions and concluding remarks, respectively.

## 2 Background

Task recognition involves classifying a human’s task action based on a set of domain relevant activities, or tasks (Wang et al., 2019). A task, *t*_*k*_, belongs to a task set *T*, for which a sequence of sensor readings *S*_*k*_ corresponds to the task. Task recognition is intended to identify a function *f* that predicts the task performed based on the sensor readings *S*_*k*_, such that the discrepancy between the predicted task $\hat{t_{k}}$ and the ground truth *t*_*k*_ is minimized. *f* does not usually take *S*_*k*_ as direct input, as the sensor readings often require a processing function *Φ* that converts the sensor readings *S*_*k*_ into a *d*-dimensional feature vector $\Phi \left(s\right)≐x\in R^{d}$ by extracting meaningful features (Wang et al., 2019). The function *f* inputs the feature vector **x** to predict the task $\hat{t_{k}}$ . Due to human’s individual differences multiple different feature vectors can be mapped to a single task. Therefore, machine learning algorithms are adopted for learning the function *f* (Lara and Labrador, 2013).

Generally, human’s tasks encompass multiple activity components: Physical movements, cognitive, visual, speech and auditory. Physical movements can be categorized based on motion granularity: 1) Gross motor, 2) Fine-grained motor, and 3) Tactile. Gross motor tasks’ physical movements displace the entire body, such as walking, running, and climbing stairs, or major portions, such as swinging an arm (e.g., Cleland et al., 2013; Chen and Xue, 2015; Allahbakhshi et al., 2020). Fine-grained motor tasks involve body extremities’ motion (i.e., wrists and fingers), such as grasping and object manipulation (e.g., Zhang et al., 2008; Fathi et al., 2011; Laput and Harrison, 2019). Tactile tasks’ physical movements result in sense of touch, such as mouse clicks, keyboard strokes, and carrying a backpack (e.g., Hsiao et al., 2015; Lee and Nicholls, 1999). Cognitive tasks use the brain to process new information, as well as recall or retrieve information from memory (e.g., Kunze et al., 2013a; Yuan et al., 2014; Salehzadeh et al., 2020). Visual task examples include identifying different objects, and reading (e.g., Bulling et al., 2010; Ishimaru et al., 2017; Srivastava et al., 2018). Speech-reliant tasks are voice articulation dependent, such as communicating over the radio (e.g., Dash et al., 2019; Abdulbaqi et al., 2020), while auditory tasks are acoustic events in the environment, such as an important announcement or emergency sounds (e.g., Stork et al., 2012; Hershey et al., 2017; Laput et al., 2018). Most existing task recognition approaches focus primarily on detecting physical gross motor and fine-grained motor tasks. However, some tasks involve little to no physical movement. Robots need a holistic understanding of tasks’ various activity components to detect them accurately.

## 3 Related work

Human task recognition has been an active field of research for more than a decade; therefore, a number of review papers with a wide range of scope and objectives exist in the literature (Lara and Labrador, 2013; Cornacchia et al., 2017; Nweke et al., 2018; Wang et al., 2019; Chen K. et al., 2021; Ramanujam et al., 2021; Bian et al., 2022; Zhang S. et al., 2022). This manuscript focuses on wearable sensor-based human task recognition; thus, this section is only focused on this domain. A brief overview of the latest task recognition surveys from the last decade is presented.

One of the earliest surveys provided an overall task recognition framework, along with its primary components incorporating wearable sensors (Lara and Labrador, 2013). The survey categorized the manuscripts based on their learning approach (supervised or semi-supervised) and response time (offline or online), and qualitatively evaluated them in terms of recognition performance, energy consumption, obtrusiveness, and flexibility. The highlighted open problems included the need for composite and concurrent task recognition. Other comprehensive surveys of task recognition using wearable sensors discussed how different task types can be detected by i) breadth of sensing modalities, ii) choosing appropriate on-body sensor locations, iii) and learning approaches (Cornacchia et al., 2017; Elbasiony and Gomaa, 2020).

The prior reviews primarily surveyed classical machine learning based approaches (e.g., Support Vector Machines and Decision Trees) for task recognition. More recent surveys reviewed manuscripts that leveraged deep learning (Nweke et al., 2018; Wang et al., 2019; Chen K. et al., 2021; Ramanujam et al., 2021; Zhang S. et al., 2022). Nweke et al. (2018) and Wang et al. (2019) presented a taxonomy of generative, discriminative, and hybrid deep learning algorithms for task recognition, while Ramanujam et al. (2021) categorized the deep learning algorithms by convolutional neural network, long short-term memory, and hybrid methods to conduct an in-depth analysis on the benchmark datasets. A comprehensive review of the deep learning challenges and opportunities was presented (Chen K. et al., 2021). Zhang S. et al. (2022) focused on the most recent cutting-edge deep learning methods, such as generative adversarial networks and deep reinforcement learning, along with a thorough analysis in terms of model comparison, selection, and deployment.

Previous task recognition surveys primarily focused on discussing algorithms using wearable sensors. An in-depth understanding of the state-of-art sensing modalities is as important as the algorithmic solutions. A recent survey categorized task recognition-related sensing modalities into five classes: mechanical kinematic sensing, field-based sensing, wave-based sensing, physiological sensing, and hybrid (Bian et al., 2022). Specific sensing modalities were presented by category, along with the strengths and weaknesses of each modality across the categorization.

Other surveys focused solely on video-based task recognition algorithms, typically using surveillance-based datasets (Ke et al., 2013; Wu et al., 2017; Zhang H.-B. et al., 2019; Pareek and Thakkar, 2021), while Liu et al. (2019) reviewed algorithms that leveraged the change in wireless signals, such as the received signal strength indicator and Doppler shift to recognize tasks. The *in situ*, potentially deconstructed (e.g., 2023 Turkey–Syria earthquake) first response HRT cannot rely on such sensors for task recognition.

Most existing literature reviews focus primarily on algorithms that detect tasks involving physical movements; thus, the reviewed algorithms are biased to only include gross and fine-grained motor task recognition methods. Compared to the prior literature reviews, this manuscript acknowledges that tasks have multiple dimensions and proposes a task taxonomy based on motion granularity (i.e., gross motor, fine-grained motor, and tactile), as well as other task component channels (i.e., visual, cognitive, auditory and speech). The manuscript’s primary contributions are summarized below.

• A comprehensive list of wearable sensor-based metrics are evaluated to assess the metrics’ ability to detect tasks within the context of the first-response HRT domain.
• A systematic review of relevant manuscripts over the years that are categorized by the seven activity components and grouped based on the machine learning methods.
• A set of criteria to evaluate the reviewed manuscripts’ ability to recognize tasks performed by first-response human teammates operating in HRTs.

The criteria developed to evaluate the metrics and algorithms may appear restrictive, as they are grounded in the first-response domain; however, the classifications provided are widely applicable for domains that involve unstructured, dynamic environments that require wearable sensors and demand a holistic understanding of a human’s task state.

## 4 Task recognition metrics

Task recognition algorithms require metrics (e.g., inertial measurements, pupil dilation, and heart-rate) to detect the tasks performed by humans. The task recognition metrics incorporated by an algorithm inform how accurately a given set of tasks and the associated activity components can be detected for a particular task domain; therefore, selecting the right set of metrics takes precedence over algorithm development. Thirty task recognition metrics were identified across the existing literature. Three criteria were developed to evaluate the metrics’ ability to detect tasks performed by first-response HRTs operating in uncertain, unstructured environments.

*Sensitivity* refers to a metric’s ability to detect tasks reliably. A metric’s sensitivity is classified as *High* if at least three citations indicate that the metric detects tasks with ≥80% accuracy, while a metric is classified as *Medium* if the task detection accuracy is ≥70% , but <80% . *Low* metric sensitivity occurs if the metric detects tasks with <70% accuracy. Metrics without sufficient citations to determine their sensitivity are classified as *Indeterminate*, and additional evidence is required to substantiate the metric’s sensitivity.

*Versatility* refers to a metric’s ability to detect tasks across different task domains. A metric’s versatility is *High* if the metric is cited for discriminating tasks in at least two or more task domains. Similarly, if the metric was used for classifying tasks belonging to only one task domain, the versatility is *Low*.

*Suitability* evaluates a metric’s feasibility to detect tasks in various physical environments (i.e., structured vs unstructured), which depends on the sensor technology for gathering the metric. Some metrics (e.g., eye gaze) can be acquired using many different sensors and technologies, but the review’s focus on disaster response encourages the use of wearable sensors over environmentally embedded sensors. A metric’s *suitability* is *conforming* if it is cited to be gathered by a *wearable* sensor that is unaffected by disturbances (e.g., sensor displacement noise, excessive perspiration, and change in lighting conditions), while *non-conforming* otherwise.

The task recognition metrics and the corresponding sensitivity, versatility, and suitability classifications are provided in Table 1. The *Activity Component* column in Table 1 indicates which component(s) (i.e., gross motor, fine-grained motor, tactile, visual, cognitive, auditory, and speech) are associated with the metric. The metrics are categorized based on their sensing properties. Each metric is evaluated based on the three evaluation criteria in order to identify the most reliable, minimal set of metrics required to recognize HRT tasks.

**TABLE 1:** Metrics evaluation overview by Sensitivity (**Sens.**), Versatility (**Verst.**), and Suitability (**Suit.**), where ⋁, ***∏***, and ⋀, represent Low, Medium, and High, respectively **(.)** indicates Indeterminate, while * indicate hypothesis predicted for the particular metric. Suitability is classified as conforming (**C**) or non-conforming (**NC**).

Category	Metrics	Sens	Verst	Suit	Activity component
Inertial	Acceleration	⋀	⋀	C	Gross
					Fine-grained
					Tactile
	Orientation	(⋀*)	⋀	C	Gross
					Fine-grained
Eye Gaze	Fixation	⋀	⋀	C	Visual
	Saccades	*∏*	⋀	C	Visual
	Scanpath	*∏*	⋁	C	Visual
	Blink rate	(⋁*)	⋀*	C	Visual
		(⋀*)	⋀*	C	Cognitive*
	Pupil dilation	(⋀*)	(⋀*)	NC	Visual*
					Cognitive*
Electro-physiological	EOG	*∏*	⋀	NC	Visual
		(⋀*)	⋀	NC	Cognitive
	sEMG	⋀	⋀	NC	Gross
					Fine-grained
		(⋀*)	⋀	NC	Tactile
	EEG	⋀	⋁	NC	Cognitive
	ECG	⋁	⋁	C	Gross
	Heart-rate	⋁	⋁	C	Gross
	Heart-rate variability	(⋀*)	(⋀*)	C	Cognitive*
Vision	Optical flow	⋀	⋀	NC	Gross
		*∏*	⋀	NC	Fine-grained
	Human-body pose	*∏*	⋀	NC	Gross
		⋀	⋀	NC	Fine-grained
	Object detection	⋁	⋀	NC	Gross
					Fine-grained
Acoustic	Spectrogram	⋀	⋀	C	Auditory
	MFCCs	⋀	⋀	C	Auditory
	Noise level	(⋁*)	(⋀*)	C	Auditory
Speech	Transcript	(∏*)	(⋁*)	C	Speech
	Keywords	(∏*)	(⋁*)	C	Speech
	Speech rate	(⋀*)	(⋀*)	C	Speech
	Voice intensity	(⋀*)	(⋀*)	C	Speech
	Voice pitch	(⋀*)	(⋀*)	C	Speech
Localization	Outdoor localization	⋁	⋀	NC	Gross
	Indoor localization	⋀	⋀	NC	Gross
					Fine-grained
Miscellaneous	Physiological	⋁	⋀	C	Gross

### 4.1 Inertial metrics

*Inertial* metrics consists of: i) linear acceleration, which measures a body region’s three-dimensional movement via an accelerometer; and ii) the body part’s three-dimensional orientation (i.e., rotation and rotational rate) using gyroscope and magnetometer. Inertial metrics are primarily used for detecting physical tasks, which includes gross motor, fine-grained motor and tactile tasks (Cleland et al., 2013; Lara and Labrador, 2013; Vepakomma et al., 2015; Cornacchia et al., 2017).

Linear acceleration is the most widely employed, and can be used as a standalone task recognition metric. Orientation is often used in combination with linear acceleration. The type and number of tasks detected by the inertial metrics can be linked to the number and placement of the sensors on the body (Atallah et al., 2010; Cleland et al., 2013). For example, inertial metric sensors to detect gross motor tasks are placed at central or lower body locations (i.e., chest, waist and thighs) (Ravi et al., 2005; Kwapisz et al., 2011; Mannini and Sabatini, 2011; Gjoreski et al., 2014), while fine-grained motor tasks require the sensor on the forearms and wrists (Zhang et al., 2008; Koskimaki et al., 2009; Min and Cho, 2011; Heard et al., 2019a), and tactile tasks place the sensors at the hand’s dorsal side and fingers (Jing et al., 2011; Cha et al., 2018; Liu et al., 2018). Linear acceleration has high sensitivity, while standalone orientation is indeterminate. The inertial metrics have high versatility and conform with suitability criteria.

### 4.2 Eye gaze metrics

*Eye gaze* metrics record the coordinates (*g*_*x*_, *g*_*y*_) of the gaze point over time. Raw eye gaze data is often processed to yield eye movement metrics representative of a human’s visual behavior and can be leveraged for task recognition. Fixations, saccades, scanpath, blink rate, and pupil dilation represent some of the important eye gaze-based metrics (Kunze et al., 2013b; Steil and Bulling, 2015; Martinez et al., 2017; Hevesi et al., 2018; Srivastava et al., 2018; Kelton et al., 2019; Landsmann et al., 2019). These metrics are commonly used for recognizing visual tasks, while some prior research have also detected cognitive tasks (Kunze et al., 2013c; Islam et al., 2021).

*Fixations* are stationary eye states during which gaze is held upon a particular location (Bulling et al., 2010), while *saccades* are the simultaneous movement of both eyes between two fixations (Bulling et al., 2010). Fixation has high sensitivity (e.g., Steil and Bulling, 2015; Kelton et al., 2019; Landsmann et al., 2019), while a saccade has medium sensitivity (e.g., Bulling et al., 2010; Ishimaru et al., 2017; Srivastava et al., 2018). Both metrics have high versatility and conform with suitability.

A *scanpath* is a fixation-saccade-fixation sequence (Martinez et al., 2017). Scanpaths have medium sensitivity (Martinez et al., 2017; Srivastava et al., 2018; Islam et al., 2021), conform with suitability, and have low versatility.

*Blink rate* represents the number of blinks (i.e., opening and closing eyelids) per unit time (Bulling et al., 2010). Blink rate is often used in conjunction with fixations and saccades to provide further context. The metric’s sensitivity is indeterminate, but it is hypothesized to have low sensitivity and is used predominantly to detect desktop or office-based visual tasks (e.g., Bulling et al., 2010; Ishimaru et al., 2014a). Prior research reviews indicate that blink rate highly correlates with the cognitive workload (Marquart et al., 2015; Heard et al., 2018a); therefore, blink rate is hypothesized to have high sensitivity toward cognitive task recognition. This metric is also hypothesized to have high versatility given its potential to be used in multiple task domains, and it conforms with suitability.

*Pupil dilation*, or pupillometry, is the change in pupil diameter. The metric’s sensitivity is indeterminate; however, hypothesized to have high sensitivity and versatility toward cognitive and visual task recognition based on its ability to reliably detect cognitive workload (Ahlstrom and Friedman-Berg, 2006; Marquart et al., 2015; Heard et al., 2018a), and its high correlation in various visual search tasks (Porter et al., 2007; Privitera et al., 2010; Wahn et al., 2016). Environmental lighting changes can significantly impact the metric’s acquisition, so it does not conform with suitability.

### 4.3 Electrophysiological metrics

The *electrophysiological* metrics refer to the electrical signals associated with various body parts (e.g., muscles, brain and eyes). These signals can be leveraged for task recognition, as they are highly correlated with tasks humans conduct. The most common electrophysiological metrics are electromyography, electrooculography, electroencephalography, and electrocardiography.

*Electromyography* measures the potential difference caused by contracting and relaxing muscle tissues. Surface-electromyography (sEMG) is a non-invasive technique, wherein electrodes placed on the skin measure the electromyography signals. A forearm positioned sEMG commonly detects fine-grained motor tasks (e.g., Koskimäki et al., 2017; Heard et al., 2019b; Frank et al., 2019), while upper limb positioned sEMG can detect gross motor tasks (e.g., Scheme and Englehart, 2011; Trigili et al., 2019). sEMG has high sensitivity and versatility for detecting gross and fine-grained motor tasks. The metric has been employed for detecting various finger and intricate hand motions (e.g., Chen et al. (2007a; b); Zhang et al. (2009)); thus, it is hypothesized to have high sensitivity for detecting tactile tasks. Finally, the metric does not conform with suitability, as sweat accumulation underneath the electrodes may compromise the sEMG sensor’s adherence to the skin, as well as the associated signal fidelity (Abdoli-Eramaki et al., 2012).

The *Electrooculography (EOG)* metric measures the potential difference between the cornea and the retina caused by eye movements. The metric has medium sensitivity for classifying visual tasks (e.g., typing, web browsing, reading and watching videos) (Bulling et al., 2010; Ishimaru et al., 2014b; Islam et al., 2021). The metric is capable of detecting cognitive tasks (e.g., Datta et al., 2014; Lagodzinski et al., 2018), but its cognitive sensitivity is indeterminate. The metric has high versatility (Datta et al., 2014; Lagodzinski et al., 2018). EOG does not conform with suitability, because it is susceptible to noise introduced by facial muscle movements (Lagodzinski et al., 2018).

*Electroencephalography (EEG)* collects electrical neurophysiological signals from different parts of the brain. EEG measures two different metrics: i) the event-related potential measures the voltage signal produced by the brain in response to a stimulus (e.g., Zhang X. et al., 2019; Salehzadeh et al., 2020); and ii) the power spectral density measures the power present in the signal spectrum (e.g., Kunze et al., 2013a; Sarkar et al., 2016). Both metrics have high sensitivity. EEG signals may be inaccurate when a human is physically active, so the metrics are best suited for detecting cognitive tasks in a sedentary environment. Therefore, the EEG metrics have low versatility. EEG signals suffer from low signal-to-noise ratios (Schirrmeister et al., 2017; Zhang X. et al., 2019), and incorrect sensor placement can create inaccuracies; therefore, EEG metrics do not conform with suitability.

*Electrocardiography* (ECG) measures the heart’s electrical activity. Standalone ECG signals are not sensitive enough to detect tasks; therefore, ECG is often used in conjunction with inertial metrics to detect gross motor tasks (Jia and Liu, 2013; Kher et al., 2013). ECG signal has low versatility, as it has been used to detect only ambulatory tasks (e.g., Jia and Liu, 2013; Kher et al., 2013), but it does conform with suitability.

The ECG signals can be used to measure two other metrics: i) *heart-rate* measures the number of heart beats per minute, while ii) *heart-rate variability* measures the variation in the heart-rate’s beat-to-beat interval. Heart-rate has low sensitivity for detecting gross motor tasks (e.g., Tapia et al., 2007; Park et al., 2017; Nandy et al., 2020) and is often used for distinguishing the humans’ intensity when performing physical tasks (Tapia et al., 2007; Nandy et al., 2020). Heart-rate has low versatility, as it can only detect ambulatory tasks. The heart-rate metric conforms with suitability if a human’s stress and fatigue levels remain constant. The heart-rate variability metric has seldom been used for task recognition (Park et al., 2017), but it is sensitive to large variations in cognitive workload (Heard et al., 2018a). Therefore, the metric is hypothesized to have high sensitivity for cognitive task recognition. The metric conforms with suitability, and is hypothesized to have high versatility.

### 4.4 Vision-based metrics

*Vision-based* metrics (e.g., optical flow, human-body pose and object detection) use videos and images containing human motions in order to infer the tasks being performed (Fathi et al., 2011; Garcia-Hernando et al., 2018; Ullah et al., 2018; Neili Boualia and Essoukri Ben Amara, 2021). These metrics are acquired via environmentally embedded cameras installed at fixed locations, or using wearable cameras mounted on a human’s shoulders, head, or chest (e.g., Mayol and Murray, 2005; Fathi et al., 2011; Matsuo et al., 2014).

Vision-based metrics detect gross and fine-grained motor tasks by enabling various computer vision algorithms [e.g., object detection, localization, and motion tracking He et al. (2016); Deng et al. (2009); Redmon et al. (2016)], which are relevant for task recognition. The metrics’ use is discouraged for the intended HRT domain, because i) environmentally embedded cameras are not readily available in unstructured domains; ii) high susceptibility to background noise from lighting, vibrations, and occlusion; iii) raise privacy concerns, and iv) computationally expensive to process (Lara and Labrador, 2013). The metrics have medium to high sensitivity, high versatility, and non-conforming with suitability.

### 4.5 Acoustic metrics

Acoustic metrics leverage the characteristic sounds in order to detect auditory events in the surrounding environment. Auditory event recognition algorithms commonly use two types of frequency-domain metrics. A *spectrogram* is a three-dimensional acoustic metric representing a sound signal’s amplitude over time at various frequencies (Haubrick and Ye, 2019). The spectrogram has high sensitivity and versatility (e.g., Laput et al., 2018; Haubrick and Ye, 2019; Liang and Thomaz, 2019). Cepstrum represents the short-term power spectrum of a sound, and is obtained by applying an inverse Fourier transformation on a sound wave’s spectrum. The *Mel Frequency Cepstral Coefficients (MFCCs)* represent the amplitudes of the resulting cepstrum on a Mel scale. The MFCC metric has high sensitivity and versatility (e.g., Min et al., 2008; Stork et al., 2012). Both metrics conform with suitability, as long as the audio is captured via a wearable microphone.

*Noise level* measures a task environment’s loudness in decibels. Noise level correlates to an increase in auditory workload (Heard et al., 2018b), but has not been used for task recognition; however, the metric is hypothesized to detect auditory events when the events are fewer (≤3) . The metric’s sensitivity, versatility, and suitability are hypothesized to be low, high, and conforming, respectively.

### 4.6 Speech metrics

Communication exchanges between human teammates can be translated into text, or a *Verbal transcript*, such that the message is captured as it was spoken. Transcripts can be generated manually (Gu et al., 2019), or using an automatic speech recognition tool [e.g., SPHINX (Lee et al., 1990), Kaldi (Povey et al., 2011), Wav2Letter++ (Pratap et al., 2019)]. The transcribed words are encoded into *n* − dimensional vectors [e.g., *GloVe* vector embeddings (Pennington et al., 2014)] to be used as inputs for detecting speech-reliant tasks (Gu et al., 2019). Representative *keywords* that are spoken more frequently can be used for detecting tasks (Abdulbaqi et al., 2020). Keywords can be detected for every utterance automatically using word-spotting (Tsai and Hao, 2019; Gao et al., 2020). Identifying keywords for each task is non-trivial and requires considerable human effort. Both transcript and keywords metrics’ sensitivity is indeterminate, but is hypothesized to be medium (Gu et al., 2019; Abdulbaqi et al., 2020). The metrics conform with suitability, provided the speech audio is obtained using a wearable microphone that minimize extraneous ambient noise. The metrics are exceptionally domain specific; therefore, their versatility is hypothesized to be low.

Several speech-related metrics (e.g., speech rate, pitch, and voice intensity) that do not rely on natural language processing have proven effective for estimating speech workload (Heard et al., 2018a; Heard et al., 2019a; Fortune et al., 2020). *Speech rate* captures verbal communications’ articulation rate by measuring the number of syllables uttered per unit time (Fortune et al., 2020). *Voice intensity* is the speech signal’s root-mean-square value, while *Pitch* is the signal’s dominant frequency over a time period (Heard et al., 2019a). These metrics have not been used for task recognition; therefore, additional evidence is required to substantiate their evaluation criteria. The metrics’ sensitivity and versatility are hypothesized to be high. The suitability criterion is conforming, assuming that the speech audio is obtained via wearable microphones.

### 4.7 Localization-based metrics

Localization-based metrics infer tasks by analyzing either the absolute or relative position of items of interest, including humans. The outdoor localization metric measures a human’s absolute location (i.e., latitude and longitude coordinates) using satellite navigation systems. This metric has low sensitivity, (Liao et al., 2007), but can support task recognition by providing context (Reddy et al., 2010; Riboni and Bettini, 2011). The metric is highly versatile and non-conforming with suitability (Lara and Labrador, 2013).

Indoor localization determines the relative position of items, including humans, relative to a known reference point in indoor environments by acquiring the change in radio signals. Radio Frequency Identification (RFID) tags and wireless modems installed at stationary locations are the standard options. The indoor localization metric infers tasks by determining humans’ location or identifying objects lying in close proximity (Cornacchia et al., 2017; Fan et al., 2019). The metric has high sensitivity and versatility. The metric requires environmentally embedded sensors; therefore, it is non-conforming for suitability.

### 4.8 Physiological metrics

Physiological metrics provide precise information about a human’s vital state. Several physiological metrics exist: i) *Galvanic skin response*, which measures the skin’s conductivity, ii) *Respiration rate*, which represents the number of breaths taken per minute, iii) *Posture Magnitude*, which measures a human’s trunk flexion (leaning forward) and extension (leaning backward) angle in degrees, and iv) *Skin temperature*, are the most commonly used physiological metrics for task recognition (Min and Cho, 2011; Lara et al., 2012). The physiological metrics have low sensitivity, as they react to activity changes with a time delay. The metrics have high versatility and conform with suitability.

## 5 Task recognition algorithms

Over one hundred task recognition algorithms across different activity components and task domains were identified and reviewed. The algorithms are evaluated using the following criteria: sensitivity, suitability, generalizability, composite factor, concurrency, and anomaly awareness. The evaluation criteria and the corresponding requirements were chosen in order to assess an algorithm’s viability for detecting tasks in a human-robot teaming domain.

### 5.1 Evaluation criteria

*Sensitivity* refers to an algorithm’s ability to detect tasks reliably. An algorithm’s sensitivity is classified as *High* if the algorithm detects tasks with ≥80% accuracy, while *Medium* if the algorithm’s accuracy is ≥70% , but <80% , and *Low* if the accuracy is <70% . The accuracy thresholds were chosen by fitting a skewed Gaussian curve on the reviewed task recognition algorithms’ accuracies.

An algorithm’s *suitability* evaluates its feasibility for detecting tasks in various physical environments. An algorithm conforms if it can detect tasks independent of the environment by incorporating wearable, reliable metrics, and is non-conforming otherwise.

*Generalizability* represents an algorithm’s ability to identify tasks across humans. The generalizability criterion depends on the achieved accuracy, given the algorithm’s validation method. An algorithm conforms if it achieves ≥80% accuracy with *leave-one-subject-out* cross-validation or *in-the-wild* validation.

The *Composite factor* criterion determines whether an algorithm can detect tasks composed of multiple atomic activities. If a detected task incorporates two or more atomic activities, then the algorithm conforms with the composite task criterion. Typically, long duration tasks that incorporate multiple action sequences per task are composite in nature.

*Concurrency* determines if the algorithm can detect tasks executed simultaneously. Concurrency has multiple forms: i) a task may be initiated prior to completing a task, such that a portion of the task overlaps with the prior task (i.e., *interleaved tasks*), and ii) multiple tasks performed at the same time (i.e., *simultaneous tasks*) (Allen and Ferguson, 1994; Liu et al., 2017). An algorithm conforms if it can detect at least one form of concurrency, and is non-conforming otherwise.

*Anomaly Awareness* determines an algorithm’s ability to detect an out-of-class task instance, which arises when an algorithm encounters sensor data that does not correspond to any of the algorithm’s learned tasks. An algorithm conforms with anomaly awareness if it can detect out-of-class instances.

Most task recognition algorithms can only detect a predefined set of atomic tasks and are unable to detect concurrent tasks or out-of-class instances (Lara and Labrador, 2013; Cornacchia et al., 2017). Thus, unless identified otherwise, the reviewed algorithms do not conform with composite factor, concurrency and anomaly awareness.

### 5.2 Overview of task recognition algorithm categories

Task recognition algorithms typically incorporate supervised machine learning to identify the tasks from the sensor data (see Section 2). These algorithms can be grouped into several categories based on feature extraction, ability to handle uncertainty, and heuristics. Three common data-driven task recognition algorithm categories exist in the literature, which are.

• *Classical machine learning* rely on features extracted from raw sensor data to learn a prediction model. Classical approaches are suitable when there is sufficient domain knowledge to extract meaningful features, and the training dataset is small.
• *Deep learning* avoids designing handcrafted features, learns the features automatically (Goodfellow et al., 2016), and is generally suitable when a large amount of data is available for training the model. Deep learning approaches leverage data to extract high-level features, while simultaneously training a model to predict the tasks.
• *Probabilistic graphical models* utilize probabilistic network structures (e.g., Bayesian Networks (Du et al., 2006), Hidden Markov Models (Chung and Liu, 2008), Conditional Random Fields (Vail et al., 2007)) to model uncertainties and the tasks’ temporal relationships, while also identifying composite, concurrent tasks.

The data-driven models’ primary limitations are that they i) cannot be interpreted easily, and ii) may require large amount of training data to be robust enough to handle individual differences across humans and generalize across multiple domains.

*Knowledge-driven* task recognition models exploit heuristics and domain knowledge to recognize the tasks using reasoning-based approaches [e.g., ontology and first-order logic (Triboan et al., 2017; Safyan et al., 2019; Tang et al., 2019)]. Knowledge-driven models are logically elegant and easier to interpret, but do not have enough expressive power to model uncertainties. Additionally, creating logical rules to model temporal relations becomes impractical when there are a large number of tasks with intricate relationships (Chen and Nugent, 2019; Liao et al., 2020).

### 5.3 Gross motor tasks

Gross motor tasks occur across multiple task categories, such as Activities of Daily Living (ADL), *fitness*, and, *industrial*. A high-level overview of the reviewed algorithms with regard to the evaluation criteria is presented by algorithm category in Table 2.

**TABLE 2:** Gross motor task recognition algorithms evaluation overview by Sensitivity (Sens.), Suitability (Suit.), Generalizability (Genr.), Composite Factor (Comp.), Concurrency (Conc.), and Anomaly Awareness (Anom.). Sensitivity is classified as Low (⋁), Medium (*∏*), or High (⋀), while other criteria are classified as conforming (C), non-conforming (NC), or requiring additional evidence (RE).

Category	Paper	Sens	Suit	Genr	Comp	Conc	Anom
Algorithm							
Classical Machine Learning							
Artificial neural network	Kher et al. (2013)	⋀	C	NC	NC	NC	NC
Decision trees	Parkka et al. (2006)	⋀	C	C	NC	NC	NC
	Tapia et al. (2007)	⋁	C	NC	NC	NC	NC
Ensemble	Nandy et al. (2020)	⋀	C	C	NC	NC	NC
k-Nearest Neighbors	Kubota et al. (2019)	⋀	NC	C	NC	NC	NC
	Ladjailia et al. (2020)	⋀	NC	RE	NC	NC	NC
Logistic regression	Lara et al. (2012)	⋀	C	NC	NC	NC	NC
Plurality voting	Ravi et al. (2005)	⋀	C	NC	NC	NC	NC
Random forest	Allahbakhshi et al. (2020)	⋀	C	C	NC	NC	NC
Recurrent neural network	Batzianoulis et al. (2017)	⋀	NC	NC	NC	NC	NC
Relevance vector machines	Jia and Liu (2013)	⋀	C	NC	NC	NC	NC
SVM	Park et al. (2017)	⋀	C	C	NC	NC	NC
	Kumar and John (2016)	⋀	NC	C	NC	NC	NC
	Schuldt et al. (2004)	⋁	NC	NC	NC	NC	NC
Deep Learning							
CNN	Chen and Xue (2015)	⋀	C	C	NC	NC	NC
	Alsheikh et al. (2016)	⋀	C	NC	NC	NC	NC
	Ignatov (2018)	⋀	C	C	NC	NC	NC
	Lee et al. (2017)	⋀	C	NC	NC	NC	NC
LSTM	Inoue et al. (2018)	⋀	C	NC	NC	NC	NC
CNN + LSTM	Fan et al. (2019)	⋀	NC	RE	NC	NC	NC
	Peng et al. (2018)	⋀	C	NC	C	NC	NC
	Chen et al. (2021b)	*∏*	C	NC	C	NC	NC
CNN + GRU	Xu et al. (2019)	⋀	C	NC	C	NC	NC
Transformer	Dirgová Luptáková et al. (2022)	⋀	C	NC	NC	C	NC
Probabilistic Graphical Model							
Bayesian network	Zhang et al. (2013)	⋁	C	NC	C	C	NC
Conditional random field	Hu et al. (2016)	⋀	NC	RE	NC	NC	NC
Gaussian mixture model	Trigili et al. (2019)	*∏*	NC	NC	NC	NC	NC
Hidden Markov model	Jalal et al. (2017)	⋀	NC	RE	NC	NC	NC
	Kolekar and Dash (2016)	*∏*	NC	NC	NC	NC	NC
Knowledge-driven							
Dynamic time warping	Ding et al. (2015)	⋀	NC	RE	NC	NC	NC
Principal component analysis	Pawar et al. (2007)	⋀	C	NC	NC	NC	NC
Trigger-based	Orr et al. (2018)	⋀	NC	RE	NC	C	NC

#### 5.3.1 Classical machine learning

Most gross motor task recognition algorithms incorporate classical machine learning using inertial metrics, often measured at central and lower body locations (Cornacchia et al., 2017), such as the chest (e.g., Li et al., 2010; Lara et al., 2012; Braojos et al., 2014), waist (e.g., Arif et al., 2014; Weng et al., 2014; Capela et al., 2015; Allahbakhshi et al., 2020), and thighs (e.g., Braojos et al., 2014; Gjoreski et al., 2014; Weng et al., 2014). Generally, inertial metrics measured at upper peripheral locations (e.g., forearms and wrists) are not suited for detecting gross motor tasks (Kubota et al., 2019).

Algorithms may also combine inertial data with physiological metrics, such as ECG, heart-rate, respiration rate, or skin temperature (e.g., Parkka et al., 2006; Jia and Liu, 2013; Kher et al., 2013; Park et al., 2017; Nandy et al., 2020). These algorithms extract time- and frequency-domain features and use conventional classifiers [e.g., Support Vector Machine (SVM) (Jia and Liu, 2013; Park et al., 2017), Decision Trees (Parkka et al., 2006; Tapia et al., 2007), Random Forest (Allahbakhshi et al., 2020; Nandy et al., 2020), or Logistic Regression (Lara et al., 2012)]. Physiological data can increase recognition accuracy by providing additional context, such as distinguishing between intensity levels [e.g., *running* and *running with weights* (Nandy et al., 2020)]. However, the metrics may also disrupt real-time task recognition, as they are not sensitive to sudden changes in physical activity. For example, incorporating heart-rate reduced performance when heart-rate remained high after performing physically demanding tasks, even when the human was lying or sitting (Tapia et al., 2007).

Classical machine learning algorithms involving vision-based metrics leverage optical flow extracted from stationary cameras for gross motor task recognition (e.g., Kumar and John, 2016; Ladjailia et al., 2020). Task specific motion descriptors derived from optical flow are used as features to train a machine learning classifier (e.g., SVM (Kumar and John, 2016) or k-Nearest Neighbors (Ladjailia et al., 2020)).

Generally, classical machine learning based gross motor task detection algorithms typically have high *sensitivity*, primarily due to the atomic and repetitive nature of gross motor tasks. These algorithms conform with *suitability* when the metrics incorporated are wearable and reliable (Ravi et al., 2005; Parkka et al., 2006; Tapia et al., 2007; Lara et al., 2012; Jia and Liu, 2013; Kher et al., 2013; Park et al., 2017; Allahbakhshi et al., 2020; Nandy et al., 2020), and are non-conforming otherwise (Schuldt et al., 2004; Kumar and John, 2016; Kubota et al., 2019; Ladjailia et al., 2020). Overall, the algorithms conform with *generalizability*, as they typically achieved high accuracy using a leave-one-subject-out cross-validation (Parkka et al., 2006; Kumar and John, 2016; Park et al., 2017; Kubota et al., 2019; Allahbakhshi et al., 2020; Nandy et al., 2020). All evaluated algorithms are non-conforming for the *concurrency*, *composite factor*, and *anomaly awareness* criteria.

#### 5.3.2 Deep learning methods

Classical machine learning algorithms require handcrafted features that are highly problem-specific, and generalize poorly across task categories (Saez et al., 2017). Additionally, those algorithms cannot represent the composite relationships among atomic tasks, and require significant human effort to select features and sensor data thresholding (Saez et al., 2017). Comparative studies indicate deep learning algorithms outperform classical machine learning when large amount of training data is available (Gjoreski et al., 2016; Saez et al., 2017; Shakya et al., 2018).

Deep learning algorithms involving inertial metrics typically require little to no sensor data preprocessing. A Convolutional Neural Network (CNN) detected eight gross motor tasks (e.g., falling, running, jumping, walking, ascending and descending a staircase) using raw acceleration data (Chen and Xue, 2015). Although inertial data preprocessing is not required, it may be advantageous in some situations. For example, a CNN algorithm transformed the *x*, *y*, and *z* acceleration into vector magnitude data in order to minimize the acceleration’s rotational interference (Lee et al., 2017). The acceleration signal’s *spectrogram*, which is a three dimensional representation of changes in the acceleration signal’s energy as a function of frequency and time, was used to train a CNN model (Alsheikh et al., 2016). Employing the spectrogram improved the classification accuracy and reduced the computational complexity significantly (Alsheikh et al., 2016).

Most recent algorithms leverage publicly available huge benchmark datasets (e.g., Roggen et al., 2010; Reiss and Stricker, 2012) to build deeper and more complex task recognition models. Deep learning algorithms combine CNNs with sequential modeling networks (e.g., Long Short-term Memory (LSTMs) (Peng et al., 2018; Chen L. et al., 2021), Gated Recurrent Units (GRUs) (Xu et al., 2019)) to detect composite gross motor tasks from inertial data. The *DEBONAIR* algorithm (Chen L. et al., 2021) incorporated multiple convolutional sub-networks to extract features based on the input metrics’ dynamicity and passed the sub-networks’ feature maps to LSTM networks to detect composite gross motor tasks (e.g., vacuuming, nordic walking, and rope jumping). The *AROMA* algorithm (Peng et al., 2018) recognized atomic and composite tasks jointly by adopting a CNN + LSTM architecture, while *InnoHAR* algorithm (Xu et al., 2019) combined the *Inception* CNN module with GRUs to detect composite gross motor tasks. Several other algorithms draw inspiration from natural language processing to detect gross motor task transitions (Thu and Han, 2021) and concurrency (Dirgová Luptáková et al., 2022) by utilizing bi-directional LSTMs and Transformers, respectively. Bi-directional LSTMs concatenate information from positive as well as negative time directions in order to predict tasks, whereas Transformers incorporate self-attention mechanisms to draw long-term dependencies by focusing on the most relevant parts of the input sequence.

RFID indoor localization is common for task recognition (e.g., Ding et al., 2015; Hu et al., 2016; Fan et al., 2019). The RFID’s *received signal strength indicator* and *phase angle* metrics are used to determine the relative distance and orientation of the tags with respect to the associated embedded environment readers (Scherhäufl et al., 2014). The two common task identification methods are: i) tag-attached, and ii) tag-free (Fan et al., 2019). *DeepTag* (Fan et al., 2019) introduced an advanced RFID-based task recognition algorithm that identified tasks in both tag-attached and tag-free scenarios. The deep learning-based algorithm used a preprocessed *received signal strength indicator* and *phase angle* information that combined a CNN with LSTMs in order to predict seven ADL tasks. Generally, the gross motor task recognition algorithms involving indoor localization have high *sensitivity*, but do not conform with *suitability* and *composite factor*.

Deep learning algorithms’ increased network complexity and abstraction alleviates most of the classical machine learning algorithms’ limitation, resulting in high sensitivity, especially when the data is abundant (Gjoreski et al., 2016; Saez et al., 2017); however, caution must be exercised to not overfit the algorithms. Deep learning algorithms can achieve high classification accuracy on multi-modal sensor data without requiring special feature engineering for each modality. For example, a hybrid deep learning algorithm trained using an 8-channel sEMG and inertial data detected thirty gym exercises (e.g., dips, bench press, rowing) (Frank et al., 2019). Deep learning algorithms rarely validate their results via leave-one-subject-out cross-validation, as in most cases the algorithms are validated by splitting all the available data randomly into training and validation datasets; therefore, the algorithms’ *generalizability* criteria either requires additional evidence, or is non-conforming.

#### 5.3.3 Probabilistic graphical models

Algorithms’ task predictions are not always accurate, as there is always some uncertainty associated with the predictions, especially when tasks overlap with one another, or share similar motion patterns (e.g., running vs running with weights). Additionally, humans may perform two or more tasks simultaneously, which complicates task identification when using classical and deep learning methods that are typically trained to predict only one task occurring at a time. Probabilistic graphical task recognition algorithms are adept at managing these uncertainties, and have the ability to model simultaneous tasks.

Probabilistic graphical models can detect gross motor tasks across various metrics [e.g., indoor localization (Hu et al., 2016), sEMG (Trigili et al., 2019), inertial (Lee and Cho, 2011; Kim et al., 2015), human-body pose (Jalal et al., 2017), optical flow (Kolekar and Dash, 2016), and object detection (Zhang et al., 2013)]. Hidden Markov Models are the most widely utilized probabilistic graphical algorithm for gross motor task recognition (e.g., Lee and Cho, 2011; Kim et al., 2015; Kolekar and Dash, 2016; Jalal et al., 2017), because Hidden Markov Model’s sequence modeling properties can be exploited for continuous task recognition (Kim et al., 2015). Hidden Markov Models also allow for modeling the tasks hierarchically (Lee and Cho, 2011), and can distinguish tasks with intra-class variances and inter-class similarities (Kim et al., 2015). Other probabilistic models [e.g., Gaussian Mixture Models (Trigili et al., 2019)] can also detect gross motor tasks. A probabilistic graphical model, the *Interval-temporal Bayesian Network*, unified Bayesian network’s probabilistic representation with interval algebra’s (Allen and Ferguson, 1994) ability to represent temporal relationships between atomic events (Zhang et al., 2013) to detect composite and concurrent gross motor tasks. The algorithm’s *sensitivity* and *generalizability* are low and non-conforming, respectively. The algorithm’s *suitability* is non-conforming, as it employed vision-based metrics. Finally, the algorithm’s *composite factor* and *concurrency* conform.

#### 5.3.4 Knowledge-driven algorithms

Gross motor rule-based task recognition algorithms incorporate template matching or thresholding to recognize tasks. A *Dynamic Time Warping* (Salvador and Chan, 2007) based algorithm detected free-weight exercises by computing the similarity between Doppler shift profiles of the reflected RFID signals (Ding et al., 2015). A principal component analysis thresholding algorithm detected ambulatory task transitions by analyzing the motion artifacts in ECG data induced by body movements (Pawar et al., 2007; 2006).

Rule-based algorithms can detect concurrent tasks, if the rules are relatively simple to derive using the sensor data. A multiagent algorithm (Orr et al., 2018) detected up to seven gross motor atomic tasks (e.g., dressing, cleaning, and food preparation). The algorithm detected up to two concurrent tasks using environmentally-embedded proximity sensors.

Rule-based systems are ideal for gross motor task detection when the sensor data is limited and can be comprehended in a relatively straightforward manner. For example, the prior rule-based multiagent algorithm detected concurrent tasks, as it was easy to form the rules using the proximity sensor data. Rule-based algorithms are unsuitable when the sensor data cannot be interpreted easily (i.e., instances of high dimensionality), or when there are a large number of tasks that have intricate relationships.

#### 5.3.5 Discussion

Most machine learning based algorithms can detect gross motor tasks reliably with acceptable suitability and generalizability when the tasks are atomic and non-concurrent with repetitive motions (e.g., Parkka et al., 2006; Chen and Xue, 2015; Park et al., 2017; Allahbakhshi et al., 2020). The human-robot teaming domain often involves composite tasks that may occur concurrently. None of the existing gross motor task detection algorithms satisfy all the required criteria for the intended domain.

The interval-temporal algorithm (Zhang et al., 2013) is the preferred approach for gross motor task detection. The algorithm can detect concurrent and composite tasks, but had low sensitivity and is non-conforming for suitability and generalizability, which can be attributed to the vision-based metrics and low-level Bayesian network’s poor classification accuracy. However, the algorithm is independent of the metrics (Zhang et al., 2013), as it operates hierarchically, utilizing the low-level atomic event predictions. Therefore, a modified version more suited to the intended domain may incorporate a classical machine learning algorithm [e.g., Random Forest (Allahbakhshi et al., 2020)] or a deep network [e.g., CNN (Chen and Xue, 2015)], depending on the amount of data available, to detect the low-level atomic tasks using inertial metrics. The interval-temporal algorithm can be used to detect the composite and concurrent gross motor tasks.

### 5.4 Fine-grained motor tasks

Fine-grained motor tasks often involve highly articulated and dexterous motions that can be performed in multiple ways. The execution and the time taken to complete the tasks differ from one human to the other. These aspects of fine-grained motor tasks can create ambiguity in the sensor data, making it difficult for the algorithms to detect such tasks; therefore, a wide range of methods adopting various sensing modalities exist for detecting fine-grained tasks accurately. The evaluation criteria for each reviewed fine-grained task recognition algorithm by algorithm category is provided in Table 3.

**TABLE 3:** Fine-grained motor task recognition algorithms’ evaluation overview.

Category	Paper	Sens	Suit	Genr	Comp	Conc	Anom
Algorithm							
Classical Machine Learning							
Ensemble	Min and Cho (2011)	⋀	C	C	C	NC	NC
k-Nearest Neighbors	Koskimaki et al. (2009)	⋀	C	C	NC	NC	NC
	Kubota et al. (2019)	⋁	NC	NC	NC	NC	NC
	Ladjailia et al. (2020)	⋁	NC	NC	NC	NC	NC
Random forest	Wang et al. (2015)	⋀	NC	C	NC	NC	NC
	Li et al. (2016a)	⋀	NC	RE	C	NC	NC
	Heard et al. (2019b)	⋁	NC	NC	C	NC	NC
SVM	Laput et al. (2015)	⋀	NC	C	NC	NC	NC
	Pirsiavash and Ramanan (2012)	⋁	NC	NC	NC	NC	NC
	Matsuo et al. (2014)	⋁	NC	NC	NC	NC	NC
	Zhang and Harrison (2015)	⋁	NC	NC	NC	NC	NC
Deep Learning							
CNN	Laput and Harrison (2019)	⋀	C	C	NC	NC	C
	Li et al. (2016b)	⋀	NC	RE	C	NC	NC
	Li et al. (2020a)	⋁	NC	NC	C	NC	NC
	Ma et al. (2016)	*∏*	NC	NC	C	NC	NC
	Castro et al. (2015)	⋀	NC	NC	NC	NC	NC
CNN + LSTM	Ullah et al. (2018)	⋀	NC	NC	NC	NC	NC
	Frank et al. (2019)	⋁	NC	NC	NC	NC	NC
LSTM	Garcia-Hernando et al. (2018)	⋁	NC	NC	NC	NC	NC
LSTM bi-directional	Zhao et al. (2018)	⋀	NC	C	C	C	NC
Residual + Attention	Mekruksavanich et al. (2022)	⋀	C	NC	C	NC	NC
	Al-qaness et al. (2022)	*∏*	C	NC	C	NC	NC
Transformer	Zhang et al. (2022b)	⋀	C	C	NC	NC	NC
Probabilistic Graphical Model							
Bayesian network	Liu et al. (2017)	⋀	C	RE	C	C	NC
	Wu et al. (2007)	⋀	NC	NC	NC	NC	NC
	Fortin-Simard et al. (2015)	⋀	NC	RE	NC	NC	NC
	Hevesi et al. (2018)	⋁	C	NC	NC	NC	NC
Conditional random field	Fathi et al. (2011)	⋁	NC	NC	C	NC	NC
Gaussian mixture model	Min et al. (2007)	⋀	C	C	NC	NC	NC
	Min et al. (2008)	⋀	C	C	NC	NC	C
Hierarchical latent SVM	Lillo et al. (2017)	⋀	NC	C	C	C	NC
Temporal memory	Zhang et al. (2008)	⋀	C	C	NC	NC	NC
Markov chains	Saguna et al. (2013)	⋀	NC	NC	C	C	NC
Probabilistic NN	Wang et al. (2012)	⋀	C	NC	NC	NC	NC
Temporal graph	Liao et al. (2020)	⋀	C	RE	C	C	NC

#### 5.4.1 Classical machine learning

Classical machine learning algorithms are suitable for detecting fine-grained motor tasks only when the tasks are short in duration, atomic, or repetitive (Heard et al., 2019a). Among the classical machine learning algorithms, k-Nearest Neighbors (e.g., Koskimaki et al., 2009; Kubota et al., 2019; Ladjailia et al., 2020), Random Forest (e.g., Wang et al., 2015; Li et al., 2016a; Heard et al., 2019b), and SVM (e.g., Min and Cho, 2011; Pirsiavash and Ramanan, 2012; Matsuo et al., 2014) are the most popular choices for fine-grained motor task detection.

Several classical machine learning algorithms use egocentric wearable camera videos for detecting ADL tasks (e.g., Pirsiavash and Ramanan, 2012; Matsuo et al., 2014; Garcia-Hernando et al., 2018). Image processing techniques (e.g., histogram of orientation or spatial pyramids) are used to detect objects and conventional machine learning algorithms recognize the tasks from the detected objects. These algorithms may also incorporate saliency detectors (Matsuo et al., 2014), or depth information (Garcia-Hernando et al., 2018) to identify the objects being manipulated. Temporal motion descriptive features from optical flow can also be used for recognizing fine-grained tasks. A k-Nearest Neighbors algorithm classified the fine-grained motor tasks (Ladjailia et al., 2020) based on a histogram constructed using the motion descriptors from optical flow.

Forearm sEMG signals can detect tasks that are difficult for a vision-based algorithm to differentiate when using the same conventional classifiers. A comparison between an sEMG [i.e., Myo armband (Sathiyanarayanan and Rajan, 2016)] and a motion capture sensor revealed that the former had higher efficacy in recognizing fine-grained motions (e.g., grasps and assembly part manipulation tasks) (Kubota et al., 2019). The classifiers with the sEMG data detected the minute variation in the muscle associated with each grasp; resulting in significantly higher recognition accuracy than using the motion capture data.

Some classical machine learning algorithms that use a single Inertial Measurement Unit (IMU) can classify fine-grained ADL tasks [e.g., eating and drinking (Zhang et al., 2008)], and assembly line activities [e.g., hammering and tightening screws Koskimaki et al. (2009)]. This approach is suitable for tasks involving a single hand (i.e., the dominant), when the number of recognized tasks is small (e.g., <5 ). For instance, five assembly line tasks were recognized using a wrist worn IMU’s acceleration and angular velocity data (Koskimaki et al., 2009). The associated time- and frequency-domain features were used to train a k-Nearest Neighbors algorithm to classify the tasks. A two-stage classification approach using acceleration metrics obtained by a wrist-worn accelerometer recognized eating and drinking (Zhang et al., 2008). However, when the tasks are composite or larger in number, the algorithms augment the IMU with different sensing modalities. Algorithms typically combine IMU with sEMG metrics measured at upper peripheral locations, such as the forearms and wrists, in order to capture highly articulated motions (Kubota et al., 2019). Increasing sensing modalities provides more task context, enabling an algorithm to discriminate a broader set of tasks.

A recent system attempted to recognize twenty-three composite clinical procedures by using metrics from two Myo armbands and statically embedded cameras (Heard et al., 2019a). The Myo’s sEMG and inertial metrics were combined with the camera’s human body pose metric to train a Random Forest classifier with majority voting. Many clinical procedures require multiple articulated fine-grained motions that range from <10s to >60s to complete. Long-duration fine-grained procedures are difficult to detect due to intra-class variability, inter-class similarity, and individual differences among participants. The video provided contextual information that improved the procedure recognition accuracy by alleviating intra-class variance and inter-class similarity.

A multi-modal framework, incorporating five inertial sensors, data gloves and a bio-signal sensor, detected eleven atomic tasks (e.g., writing, brushing, typing) and eight composite tasks (e.g., exercising, working, meeting) (Min and Cho, 2011). A hybrid ensemble approach combined classifier selection and output fusion. The sensors’ inputs were initially recognized by a Naive Bayes selection module. The selection module’s task probabilities chose a set of task-specific SVM classifiers that fused their predictions into a matrix in order to identify the tasks.

Classical machine learning algorithms’ classification accuracies range between 45% and 65%; thus, they generally have low *sensitivity*. The algorithms’ *suitability* criterion depend on the metrics employed. The *composite factor* and *generalizability* criteria also vary across algorithms, as they depend on the tasks detected and the validation methodology. Overall, most algorithms are non-conforming for the *concurrency* and *composite* factors, making them unsuitable for detecting fine-grained motor tasks for the intended HRT domain.

#### 5.4.2 Deep learning

The ambiguous, convoluted sensor data from fine-grained motor tasks causes the feature engineering and extraction to be laborious. Deep learning algorithms overcome this limitation by automating the feature extraction process. There are three different types of deep learning algorithms for fine-grained motor task recognition: i) Convolutional, ii) Recurrent, and iii) Hybrid. Convolutional algorithms typically incorporate only CNNs to learn the spatial features from sensor data for each task and distinguish them by comparing the spatial patterns (e.g., Castro et al., 2015; Li et al., 2016b; Ma et al., 2016; Laput and Harrison, 2019; Li L. et al., 2020). Recurrent algorithms detect the tasks by capturing the sequential information present in the sensor data, typically using memory cells (e.g., Garcia-Hernando et al., 2018; Zhao et al., 2018). Hybrid algorithms extract spatial features and learn the temporal relationships simultaneously by combining convolutional and recurrent networks (Ullah et al., 2018; Fan et al., 2019; Frank et al., 2019).

Deep learning algorithms using egocentric videos from wearable cameras combine object detection with task recognition. A CNN with a late fusion ensemble predicted the tasks from a chest-mounted wearable camera (Castro et al., 2015) by incorporating relevant contextual information (e.g., time and day of the week) to boost the classification accuracy. Two separate CNNs were combined together to recognize objects of interest and hand motions (Ma et al., 2016). The networks were fine tuned jointly using a triplet loss function to recognize fine-grained ADL tasks with medium to high *sensitivity*.

Analyzing changes in body poses spatially and temporally can provide important cues for fine-grained motor task recognition (Lillo et al., 2017). An end-to-end CNN network exploited camera images for estimating fifteen upper body joint positions (Neili Boualia and Essoukri Ben Amara, 2021). The estimated joint positions permitted discriminating features to recognize tasks. The CNN architecture had two levels: i) fully-convolutional layers that extracted the salient feature, or heat maps, and ii) fusion layers that learned the spatial dependencies between the joints by concatenating the convolutional layers. The CNN-estimated joint positions served as input to train a multi-class SVM that predicted twelve ADL tasks with high *sensitivity*.

Hybrid deep learning algorithms are becoming increasingly popular for task recognition across metrics (Ullah et al., 2018; Fan et al., 2019; Frank et al., 2019). An optical flow-based algorithm (Ullah et al., 2018) leveraged deep learning to extract temporal optical flow features from the salient frames, and incorporated a multilayer LSTM to predict the tasks using the temporal optical flow features. Another hybrid deep learning algorithm (Frank et al., 2019) trained on the sEMG and inertial metrics detected assembly tasks. The algorithm’s CNN layers extracted spatial features from the merits at each timestep, while the LSTM layers learned how the spatial features evolved temporally. Hybrid algorithms can provide excellent expressive and predictive capabilities; however, these algorithms’ performance relies heavily on the size of training dataset (Frank et al., 2019).

A CNN-based algorithm incorporated inertial metrics from an off-the-shelf smartwatch to detect twenty-five atomic tasks (e.g., operating a drill, cutting paper, and writing) (Laput and Harrison, 2019). A Fourier transform was applied to the acceleration data to obtain the corresponding spectrograms. The CNN identified the spatial-temporal relationships encoded in the spectrograms by generating distinctive activation patterns for each task. The algorithm also rejected (i.e., detected) unknown instances.

Deep learning algorithms can recognize concurrent and composite fine-grained motor tasks directly from raw sensor data using complex network architectures, provided sufficient data is available (Ordóñez and Roggen, 2016; Zhao et al., 2018). Human task trajectories are continuous in that the current task depends on both past and future information. A deep residual bidirectional LSTM algorithm (Zhao et al., 2018) detected the *Opportunity* dataset’s composite tasks by incorporating information from positive as well as negative time directions. The dataset contains five composite ADLs (e.g., relaxation, preparing coffee, preparing breakfast, grooming, cleaning), involving a total number of 211 atomic events (e.g., walk, sit, lying, open doors, reach for an object). Several metrics, including acceleration and orientation of various body parts, and three-dimensional indoor position were gathered.

Recent deep learning algorithms leverage attention mechanisms to model long-term dependencies from inertial data (Al-qaness et al., 2022; Zhang Y. et al., 2022; Mekruksavanich et al., 2022). The *ResNet-SE* algorithm (Mekruksavanich et al., 2022) classified composite fine-grained motor tasks on three publicly available datasets. The algorithm incorporated residual networks to address loss degradation, followed by a squeeze-and-excite attention function to modulate the relevance of each residual feature map. The *Multi-ResAtt* algorithm (Al-qaness et al., 2022) incorporated residual networks to process inertial metrics from IMUs distributed over different body locations, followed by bidirectional GRUs with attention mechanism to learn time-series features.

Generally, deep learning algorithms are highly effective at detecting atomic fine-grained motor tasks, but their ability to detect composite and concurrent tasks reliably is indeterminate. The latter may be due to insufficient ecologically-valid composite, concurrent task recognition datasets available publicly. Utilizing generative adversarial networks (Wang et al., 2018; Li X. et al., 2020) to expand datasets by producing synthetic sensor data may alleviate the issue.

#### 5.4.3 Probabilistic graphical models

Bayesian networks are the most common probabilistic graphical models for fine-grained motor task detection (e.g., Wu et al., 2007; Fortin-Simard et al., 2015; Liu et al., 2017; Hevesi et al., 2018), followed by Gaussian Mixture Models (e.g., Min et al., 2007; Min et al., 2008). Many such algorithms augment the inertial data with a different sensing modality (e.g., Min et al., 2007; Wu et al., 2007; Min et al., 2008; Hevesi et al., 2018) in order to provide more task context, which enables discriminating a broader set of tasks. Recognition of up to three day-to-day early morning tasks (Min et al., 2007) augmented with a microphone, resulted in the recognition of six tasks (Min et al., 2008). The intended HRT domain requires multiple sensors to detect tasks belonging to different activity components, although adding new modalities arbitrarily may deteriorate the classifier performance (Frank et al., 2019).

Hierarchical graphical models detect composite tasks by decomposing them into a set of smaller classification problems. Fathi et al.’s (2011) meal preparation task detection algorithm decomposed hand manipulations into numerous atomic actions, and learned tasks from a hierarchical action sequence using conditional random fields. Another hierarchical model that operated at three levels of abstraction detected composite, concurrent tasks using body poses (Lillo et al., 2017).

Identifying the causality (i.e., action and reaction pair) between two events allows for easier human interpretation, and for modeling far more intricate temporal relationships (Liao et al., 2020). A graphical algorithm incorporated the Granger-causality (Granger, 1969; 1980) test for uncovering cause-effect relationships among atomic events (Liao et al., 2020). The algorithm employed a generic Bayesian network to detect the atomic events. A temporal causal graph was generated via the Granger-causality test between atomic events. Each graph represented a particular task instance. The graph nodes represented the atomic events and directed links with weights represented the cause-effect relationships between the atomic events. An artificial neural network is trained using these graphs as inputs to predict the composite, concurrent tasks. The algorithm was evaluated on the *Opportunity* (Roggen et al., 2010) and *OSUPEL* (Brendel et al., 2011) datasets, indicating that the algorithm is independent of the metrics.

Overall, probabilistic graphical models typically have high *sensitivity* for detecting fine-grained motor tasks. The algorithms, especially hierarchical (e.g., Lillo et al., 2017) and the Granger-causality based temporal graph (Liao et al., 2020), are independent of the metrics due to data abstraction; therefore, their *suitability* is classified as conforming. Most task recognition algorithms are susceptible to individual differences (see Table 3). Even those that conform with generalizability may experience a significant decrease in accuracy when classifying an unknown human’s data (Laput and Harrison, 2019); thus, the *generalizability* criterion requires additional evidence. Algorithms can only identify tasks reliably for humans on which they were trained, suggesting that online and self-learning mechanisms are needed to accommodate new humans (Wang et al., 2012). The *composite factor* and *concurrency* vary across algorithms, but are non-conforming overall. The *anomaly awareness* criterion is classified as non-conforming, as most probabilistic graphical models do not detect out-of-class tasks.

#### 5.4.4 Discussion

Classical machine learning algorithms are unreliable for detecting fine-grained motor tasks due to poor sensitivity and generalizability. Deep learning algorithms can detect the atomic fine-grained motor tasks reliably, but not composite, concurrent tasks. Moreover, deep learning typically requires a large number of parameters, very large datasets and can be difficult to train (Lillo et al., 2017). Deep learning’s automatic feature learning capability prohibits exploiting explicit relationships among tasks and semantic knowledge, making it difficult to detect composite, concurrent fine-grained motor tasks. Probabilistic graphical models offer some suitable alternatives; however, none of the existing algorithms satisfy all the required criteria for the intended domain.

The Granger-causality based temporal graph algorithm (Liao et al., 2020) and the three-level hierarchical algorithm (Lillo et al., 2017) are the most suitable for fine-grained motor task detection given all the other algorithms. Both algorithms have high sensitivity and can detect concurrent and composite tasks. The Granger-causality algorithm conforms with suitability, but requires additional evidence to substantiate its generalizability. The hierarchical algorithm conforms with generalizability, but is non-conforming with suitability, as it employed a vision-based system for estimating human-body pose metric. However, the metric can be estimated using a series of inertial motion trackers (Faisal et al., 2019); therefore, a human-robot teaming domain friendly version of both algorithms can be developed theoretically.

### 5.5 Tactile tasks

Tactile interaction occurs when humans interact with objects around them (e.g., keyboard typing, mouse-clicking and finger gestures). Individual classifications for each tactile task algorithm by its category are provided in Table 4.

**TABLE 4:** Tactile task recognition algorithms’ evaluation overview.

Category	Paper	Sens	Suit	Genr	Comp	Conc	Anom
Algorithm							
Classical Machine Learning							
Decision trees	Jing et al. (2011)	⋀	NC	C	NC	NC	NC
Ensemble	Cha et al. (2018)	⋀	C	C	NC	NC	NC
SVM	Hsiao et al. (2015)	⋀	NC	RE	NC	NC	NC
Voting	Sha et al. (2020)	⋀	NC	NC	NC	NC	NC
Deep Learning							
CNN	Côté-Allard et al. (2019)	⋀	NC	NC	NC	NC	NC
CNN + LSTM	Rahimian et al. (2020)	⋀	NC	NC	NC	NC	NC
Probabilistic Graphical Model							
Gaussian mixture model	Ju and Liu (2014)	⋀	NC	C	NC	NC	NC
Hidden Markov model	Zhang et al. (2011)	⋀	NC	NC	NC	NC	NC
Naive bayes classifier	Chen et al. (2007b)	⋀	NC	NC	NC	NC	NC
	Chen et al. (2007a)	⋀	NC	NC	NC	NC	NC

#### 5.5.1 Classical machine learning

Most classical machine learning algorithms incorporate inertial metrics measured at the fingers or dorsal side of the hand. These approaches typically detect finger gestures and keystrokes depending on the measurement site (e.g., Jing et al., 2011; Cha et al., 2018; Liu et al., 2018; Sha et al., 2020). Several of these approaches use multiple ring-like accelerometer device worn on the fingers (e.g., Jing et al., 2011; Zhou et al., 2015; Sha et al., 2020). The time- and frequency-domain features (e.g., minimum, maximum, standard deviation, energy, and entropy) extracted from the acceleration signals were used to train classical machine learning algorithms (e.g., decision tree classifier and majority voting) to detect finger gestures (e.g., finger rotation and bending) and keystrokes. Although these approaches incorporated inertial metrics, none conform with *suitability* due to lack of reproducibility (i.e., the ring-like sensor is not commercially available) and wearing a ring-like device may hinder humans’ dexterity, impacting task performance negatively.

Inertial metrics from the dorsal side of the hand detected seven office tasks (e.g., keyboard typing, mouse-clicking, writing) (Cha et al., 2018). Time- and frequency-domain features extracted from the acceleration signals were used to train an ensemble classifier. The algorithm achieved high accuracy (>90%) in an in-the-wild evaluation. Most misclassifications occurred during transitions between tasks, implying that inertial-based tactile task recognition may be susceptible to task transitions due to signal variations. The high error rates during transitions can lead to lower classification accuracy, especially when tasks switch frequently.

Classical machine learning algorithms’ generally have high *sensitivity*. The algorithms’ are typically non-conforming for the *suitability* criterion, as many supporting research efforts focus on developing and validating new sensor technology for sensing tactility, rather than detecting tactile tasks (e.g., Iwamoto and Shinoda, 2007; Ozioko et al., 2017; Kawazoe et al., 2019). The *generalizability* criteria also vary across algorithms, as they depend on the validation methodology. Finally, the algorithms are non-conforming for the *concurrency* and *composite* factors, making them unsuitable for detecting tactile tasks for the intended HRT domain.

#### 5.5.2 Deep learning

Several publicly available sEMG-based hand gesture datasets (e.g., Atzori et al., 2014; Amma et al., 2015; Kaczmarek et al., 2019) support deep learning algorithms to detect tactile hand gestures (e.g., Côté-Allard et al., 2019; Rahimian et al., 2020). A hybrid deep learning model consisting of two parallel paths (i.e., one LSTM path and one CNN path) was developed (Rahimian et al., 2020). A fully connected multilayer fusion network combined the outputs of the two paths to classify the hand gestures.

Recognizing tactile tasks is an under-developed area of research, as the tasks are nuanced and often overshadowed by fine-grained motor tasks. Generally, deep learning algorithms have high *sensitivity*; however, the incorporated sEMG metrics with a random dataset split for validation cause them to not conform with the *suitability* and *generalizability* criteria.

#### 5.5.3 Probabilistic graphical model

Probabilistic graphical models for tactile task recognition typically involve simple algorithms (e.g., Hidden Markov Models (Zhang et al., 2011), Gaussian Mixture Models (Ju and Liu, 2014), and Bayesian Networks (Chen et al., 2007a; b)) when compared to the prior gross motor and fine-grained motor sections (see Sections 5.3.3 and 5.4.3), as the tasks detected are inherently atomic (e.g., hand and finger gestures). sEMG signals are one of the most frequently used metrics for detecting hand and finger gestures (e.g., Chen et al., 2007b; Zhang et al., 2011; Côté-Allard et al., 2019; Rahimian et al., 2020). Chen et al.’s (2007b) gesture recognition algorithm pioneered the use of sEMG signals. Twenty-five hand gestures (i.e., six wrist actions and seventeen finger gestures) were detected using a 2-channel sEMG placed on the forearm. A Bayesian classifier was trained using the mean absolute value and autoregressive model coefficients extracted from the sEMG. The algorithm was extended to include two accelerometers, one placed on the wrist and the other placed on the dorsal side of the hand (Chen et al., 2007a).

Overall, probabilistic graphical models also tend to have high *sensitivity* for detecting tactile tasks. Most algorithms are susceptible to individual differences and incorporate sEMG metrics; thus, the algorithms are non-conforming for the *suitability* and *generalizability* criteria. Additionally, the algorithms are non-conforming for the *concurrency* and *composite* factors, as the evaluated tactile tasks are inherently atomic.

#### 5.5.4 Discussion

All data-driven algorithms can detect tactile tasks with >80% accuracy (Chen et al., 2007a; Jing et al., 2011; Zhang et al., 2011; Ju and Liu, 2014; Hsiao et al., 2015; Cha et al., 2018; Rahimian et al., 2020; Sha et al., 2020) primarily because the detected tasks (i.e., finger and hand gestures) were atomic; therefore, the algorithms have high *sensitivity*. Except for the office-based tactile task classifier (Cha et al., 2018), none of the existing algorithms conform with *suitability*, because either the sensors incorporated were commercially unavailable for reproducibility, or the metrics employed were unreliable. All the algorithms are non-conforming with the *concurrency* and *composite factor* criteria, because tactile tasks are rarely composite or concurrent. Finally, none of the algorithms detect out-of-class instances; therefore, they do not conform with *anomaly awareness*. A recommended tactile task detection algorithm to support the intended domain is the interval-temporal algorithm (Zhang et al., 2013), or the Granger-causality based temporal graph (Liao et al., 2020) with the inclusion of inertial metrics measured at the dorsal side of the hand to capture the tactile component, along with the fine-grained motor component.

### 5.6 Visual tasks

Eye movement is closely associated with humans’ goals, tasks, and intentions, as almost all tasks performed by humans involve visual observation. This association makes oculography a rich source of information for task recognition. Fixation, saccades, blink rate, and scanpath are the most commonly used metrics for detecting visual tasks (Bulling et al., 2010; Martinez et al., 2017; Srivastava et al., 2018), followed by EOG potentials (Ishimaru et al., 2014b; Ishimaru et al., 2017; Lu et al., 2018). Visual tasks typically occur in *office or desktop-based* environments, where the participants are sedentary. The classifications of the reviewed visual task recognition algorithms are presented by algorithm category in Table 5.

**TABLE 5:** Visual task recognition algorithms’ evaluation overview.

Category	Paper	Sens	Suit	Genr	Comp	Conc	Anom
Algorithm							
Classical Machine Learning							
Auto-context model	Martinez et al. (2017)	⋀	C	RE	NC	NC	NC
Decision trees	Landsmann et al. (2019)	⋀	C	C	NC	NC	NC
	Ishimaru et al. (2014a)	⋀	C	NC	NC	NC	NC
k- Nearest Neighbors	Ishimaru et al. (2014b)	*∏*	NC	NC	NC	NC	NC
Random Forest	Srivastava et al. (2018)	*∏*	C	NC	NC	NC	NC
SVM	Kelton et al. (2019)	⋀	C	NC	NC	NC	NC
	Bulling et al. (2010)	*∏*	C	NC	NC	NC	NC
	Lu et al. (2018)	⋀	NC	C	NC	NC	NC
Deep Learning							
CNN	Islam et al. (2021)	⋁	NC	NC	NC	NC	NC
CNN + LSTM	Ishimaru et al. (2017)	⋁	NC	NC	NC	NC	NC
Graph CNN	Lan et al. (2020)	⋁	C	NC	NC	NC	NC
Encoder-Decoder	Meyer et al. (2022)	⋀	C	C	NC	NC	NC

#### 5.6.1 Classical machine learning

Classical machine learning using eye gaze metrics (e.g., saccades, fixation, and blink rate) for visual task recognition was pioneered by Bulling et al. (2010). Statistical features (e.g., mean, max, variance) extracted from the gaze metrics, as well as the character-based representation to encode eye movement patterns, were used to train a SVM classifier to detect five office-based tasks. The algorithm’s primary limitation is that the classification is provided at each time instance *t* independently and does not integrate long-range contextual information continuously (Martinez et al., 2017). A temporal contextual learning algorithm, the *Auto-context* model, overcame this limitation by including the past and future decision values from the discriminative classifiers (e.g., SVM and k-Nearest Neighbors) recursively until convergence (Martinez et al., 2017).

Low-level eye movement metrics (e.g., saccades and fixations) are versatile and easy to compute, but are vulnerable to overfitting, whereas high-level metrics (e.g., Area-of-Focus) may offer better abstraction, but requires domain and environment knowledge (Srivastava et al., 2018). These limitations can be mitigated by exploiting low-level metrics to yield *mid-level* metrics that provide additional context. The mid-level metrics were built on intuitions about expected task relevant eye movements. Two different mid-level metrics were identified: *shape-based pattern* and *distance-based pattern* (Srivastava et al., 2018). The shape-based pattern metrics were based on encoding different combinations of saccade and scanpath, while distance-based pattern metrics were generated using consecutive fixations. The low- and mid-level metrics were combined to train a Random Forest classifier to detect eight office-based tasks, including five desktop-based tasks and three software engineering tasks.

Various algorithms were developed focused solely on detecting reading tasks using classical machine learning (e.g., Kelton et al., 2019; Landsmann et al., 2019). The complexity of the reading task varied across algorithms. Reading detection can be as rudimentary as classifying active reading or not (Landsmann et al., 2019), or as complex as distinguishing between reading thoroughly vs skimming text (Kelton et al., 2019).

Based on feature mining, existing reading detection algorithms can be categorized into two methods: i) Global methods that mine eye movement metrics over an extended period (>30s) to build a reading detector (e.g., Bulling et al., 2010; Ishimaru et al., 2017; Martinez et al., 2017; Srivastava et al., 2018), and ii) Local methods that extract the metrics within a narrow temporal window (<3s) (e.g., Kollmorgen and Holmqvist, 2007; Biedert et al., 2012). Global methods result in better accuracy, but do not detect reading in real-time, due to longer window sizes, while local methods allow for (near) real-time reading detection, but have low accuracy (Kelton et al., 2019).

Classical machine learning algorithms (e.g., Bulling et al., 2010; Ishimaru et al., 2014b; Martinez et al., 2017; Srivastava et al., 2018; Landsmann et al., 2019) have medium to high sensitivity. Most algorithms conform with the *suitability* criterion, while rarely conforming with the *generalizability* criterion. All algorithms are non-conforming for the *concurrency* and *composite* factors, making them unsuitable for the intended HRT domain.

#### 5.6.2 Deep learning

Recent deep learning algorithms leverage CNNs to detect visual tasks directly using raw 2D gaze data obtained via wearable eye trackers. *GazeGraph* (Lan et al., 2020) algorithm converted 2D eye gaze sequence into a spatial-temporal graph representation that preserved important eye movement details, but rejected large irrelevant variations. A three-layered CNN trained on this representation detected various desktop and document reading tasks. An encoder-decoder based convolutional network detected seven mixed physical and visual tasks by combining 2D gaze data with head inertial metrics (Meyer et al., 2022).

Several other algorithms apply deep learning techniques using EOG potentials to detect reading task (Ishimaru et al., 2017; Islam et al., 2021). Two deep networks, a CNN and a LSTM, were developed to recognize reading in a natural setting (i.e., outside of the laboratory). Three metrics (i.e., blink rate, 2-channel EOG signals, and acceleration) from wearable EOG glasses were used to train the deep learning models.

Obtaining datasets at a large scale is difficult, due to high annotation costs and human effort, while lack of labeled data inhibits deep learning methods’ effectiveness. A sample efficient, *self-supervised CNN* detected reading task (Islam et al., 2021) using less labeled data. The self-supervised CNN employed a “pretext” task to bootstrap the network before training it for the actual target task. Three reading tasks (i.e., reading English documents, reading Japanese documents, both horizontally and vertically), as well as a no reading class, were detected by the self-supervised network. The pretext task recognized the transformation (i.e., rotational, translational, noise addition) applied to the input signal. The pretext pre-training phase initialized the network with good weights, which were fine-tuned by training the network on the target task (i.e., reading detection task) dataset.

The deep learning algorithms (e.g., Ishimaru et al., 2017; Lan et al., 2020; Islam et al., 2021) typically tend to have low sensitivity. Further, the EOG deep learning algorithms do not conform with *suitability*, as the employed metrics are unreliable, as it is susceptible to noise introduced by facial muscle movements (Lagodzinski et al., 2018). These limitations discourage the use of deep learning for visual task recognition.

#### 5.6.3 Discussion

None of the existing algorithms detected visual tasks within the targeted HRT context. The two classical machine learning algorithms: i) *Auto-context* model (Martinez et al., 2017) and ii) Srivastava et al.’s (2018) algorithm appear to be more appropriate for detecting visual tasks. Both algorithms had >70% accuracies across a range of visual tasks and employed eye gaze metrics; thus, conforming with *suitability* and partially with *sensitivity*. The algorithms’ *generalizability* criterion requires additional evidence, as the former’s validation scheme is unclear, while the latter does not have sufficient accuracy. None of the reviewed algorithms conform with the *concurrency* and *composite factor* criteria. The encoder-decoder algorithm (Meyer et al., 2022) is also a viable alternative, as it achieved >80% with leave-one-subject-out cross validation using eye gaze metrics.

### 5.7 Cognitive tasks

Cognition describes mental processes, including reasoning, awareness, perception, knowledge, intuition, and judgment (Lagodzinski et al., 2018), as such, most tasks require some cognitive capability. For instance, although tasks, such as reading, writing, watching videos predominantly involve visual, fine-grained motor, or tactile components, they also entail a cognitive component. Therefore, it is impractical to disregard the cognitive task elements, but classifying all such tasks as cognitive is also infeasible. Thus, only those algorithms that explicitly mention identifying the tasks’ cognitive aspect are reviewed. The evaluation of the reviewed cognitive task recognition algorithms is presented in Table 6.

**TABLE 6:** Cognitive task recognition algorithms’ evaluation overview.

Category	Paper	Sens	Suit	Genr	Comp	Conc	Anom
Algorithm							
Classical Machine Learning							
Decision trees	Ishimaru et al. (2014a)	⋀	C	NC	NC	NC	NC
Ensemble	Grana et al. (2020)	⋀	NC	NC	NC	NC	NC
k-Nearest Neighbors	Kunze et al. (2013a)	*∏*	NC	NC	NC	NC	NC
SVM	Lagodzinski et al. (2018)	⋀	NC	NC	NC	NC	NC
	Datta et al. (2014)	⋀	NC	NC	NC	NC	NC
Deep Learning							
CNN	Zhang et al. (2019b)	⋀	NC	NC	NC	NC	NC
	Sarkar et al. (2016)	⋀	NC	C	NC	NC	NC
CNN + LSTM	Salehzadeh et al. (2020)	⋀	NC	NC	NC	NC	NC

#### 5.7.1 Classical machine learning

EEG potentials, obtained by placing non-invasive electrodes on humans’ scalp, are the primary electrophysiological metrics used to detect cognitive tasks. Features (e.g., amplitude and power spectral density) extracted from the EEG frequency bands (i.e., alpha (8–12 Hz), beta (13–30 Hz), theta (4–8 Hz), and delta ( < 4 Hz)) can be used to train classical machine learning algorithms (Mostow et al., 2011; Kunze et al., 2013b; Grana et al., 2020). Reading is the most widely detected cognitive task using EEG sensing. A k-Nearest Neighbors classifier distinguished between reading and non-reading tasks (e.g., drawing, watching a video and listening to music), as well as distinguishing reading different kinds of document, using a wearable EEG sensor (Kunze et al., 2013c).

EOG is the other electrophysiological metric employed for detecting cognitive tasks (Datta et al., 2014; Lagodzinski et al., 2018). The efficacy of different EOG features in detecting cognitive tasks was investigated (Datta et al., 2014). Three features (i.e., adaptive autoregressive parameters, wavelet coefficients and Hjorth parameters) were extracted from a laboratory-developed two-channel EOG signal acquisition device. These features were used independently and in combinations to train a SVM classifier to detect eight cognitive tasks (e.g., reading, writing, copying a text, web browsing, watching a video, playing an online game, and word search).

Several other algorithms combine data from multiple sensing modalities to improve cognitive task recognition accuracy (Ishimaru et al., 2014a; Lagodzinski et al., 2018; Grana et al., 2020). For example, combining blink rate and head motion by fusing the eye gaze data with acceleration data improved a decision tree classifier’s accuracy in detecting four cognitive tasks (e.g., reading, solving a math problem, watching a video and talking) (Ishimaru et al., 2014a). The *Codebook* algorithm recognized six cognitive tasks (e.g., reading a printed page, watching a video, engaging conversation, writing handwritten notes and sorting numbers) by clustering the subsequences sampled from a data sequence based on similarity (Lagodzinski et al., 2018). The resulting cluster centers act as the set of codewords (i.e., codebook). A SVM classifier was trained to classify the histogram reflecting the codeword frequency to predict the tasks.

Classical machine learning algorithms typically have high *sensitivity*; however, the incorporated metrics (i.e., EOG and EEG) are unreliable and cannot accommodate individual differences. Therefore, the algorithms’ *suitability* and *generalizability* are non-conforming. The *composite factor* and *concurrency* are non-conforming as well.

#### 5.7.2 Deep learning

Recent deep learning advances facilitate detecting cognitive tasks using EEG potentials acquired from off-the-shelf, wireless, wearable EEG devices. Most EEG wearable devices (e.g., EMOTIV, 2021; MUSE, 2021; Neurosky, 2021) record prefrontal EEG signals, which are correlated to a human’s intellectual, emotional and cognitive states (Salehzadeh et al., 2020). A deep EEG network detected three cognitive tasks (e.g., reading, speaking, and watching a video) using data collected from a wearable EEG sensor’s (MUSE, 2021) two prefrontal EEG channels (Salehzadeh et al., 2020). The hybrid deep learning algorithm incorporated a CNN to populate the feature maps from raw EEG potentials, followed by a LSTM network for modeling the temporal state of the EEG feature maps. Most existing EEG-based algorithms focus on application-specific classification algorithms, which may not translate to other domains. A transferable EEG-based cognitive task recognition algorithm that can adaptively support varying EEG channels as input and operate on a wide range of cognitive applications was developed (Zhang X. et al., 2019). The algorithm combined deep reinforcement learning with an attention mechanism to extract robust and distinct deep features.

Detecting cognitive tasks with fewer EEG sensors in an unconstrained, natural environment is a challenging task, due to low signal-to-noise ratio, lack of baseline availability, change of baseline due to domain environment and individual differences, as well as uncontrolled mixing of various tasks (Sarkar et al., 2016). A deep learning algorithm (Sarkar et al., 2016) revealed that the backward sensor selection (Bishop and Nasrabadi, 2006) technique can reduce the sensor suite significantly (i.e., from nine probes to three) without compromising accuracy. Two deep neural networks, a deep belief network and a CNN, were trained using the EEG power spectral density to distinguish between listening and watching tasks.

Similar to the classical machine learning approaches, the deep learning algorithms also tend to have high *sensitivity*, but incorporate EEG metrics that suffer from low-signal-to-noise and individual differences; therefore, the algorithms’ *suitability* and *generalizability* are non-conforming. Additionally, none of reviewed algorithms conform with the *concurrency* and *composite factor*.

#### 5.7.3 Discussion

Generally, cognitive tasks can be classified with >80% accuracy; therefore, the algorithms’ typically have high *sensitivity*. Excluding Ishimaru et al.’s (2014a) decision tree classifier, none of the other algorithms conform with suitability, as the metrics employed were EEG or EOG. Thus, none of the discussed algorithms are appropriate for detecting cognitive tasks for the intended HRT domain. Given (a) that cognitive and visual tasks are closely associated, and (b) the efficacy of multimodality sensing (Ishimaru et al., 2014a), it is hypothesized that a classical machine learning algorithm that incorporates metrics, such as pupil dilation, blink latency and blink rate, as well as heart-rate variability will be viable for detecting cognitive tasks.

### 5.8 Auditory tasks

Auditory event recognition involves identifying characteristic ambient sounds in order to detect tasks in an environment (Mesaros et al., 2017; Laput et al., 2018). Auditory event recognition algorithms typically use microphone sensor(s) to detect the sound events. Individual classifications for each auditory event detection algorithm by its category are provided in Table 7.

**TABLE 7:** Auditory task recognition algorithms’ evaluation overview.

Category	Paper	Sens	Suit	Genr	Comp	Conc	Anom
Algorithm							
Classical Machine Learning							
Random Forest	Stork et al. (2012)	⋀	NC	C	NC	NC	NC
SVM	Yatani and Truong (2012)	*∏*	NC	NC	NC	NC	NC
Deep Learning							
CNN	Laput et al. (2018)	⋀	C	C	NC	NC	NC
	Hershey et al. (2017)	⋀	RE	RE	NC	NC	NC
	Liang and Thomaz (2019)	⋁	NC	NC	NC	NC	NC
	Haubrick and Ye (2019)	*∏*	C	NC	NC	C	NC
	Salamon and Bello (2017)	*∏*	RE	NC	NC	NC	NC

#### 5.8.1 Classical machine learning

Auditory event detection algorithms that incorporate MFCCs typically use classical machine learning (e.g., Stork et al. (2012) (Yatani and Truong, 2012)), while deep learning algorithms incorporate spectrograms (e.g., Hershey et al., 2017; Laput et al., 2018; Haubrick and Ye, 2019; Liang and Thomaz, 2019). A Random Forest based voting algorithm, *Non-Markovian Ensemble Voting*, used MFCCs to recognize characteristic sounds produced by twenty-two ADL tasks (Stork et al., 2012). The predictions were refined over time by collecting consensus via voting from the past and future predictions.

A wearable acoustic sensor, *BodyScope*, worn around the neck classified several ADL tasks (Yatani and Truong, 2012). The *BodyScope* sensor contained a microphone surrounded by a stethoscope chest piece for sound amplifications in order to exploit the sounds that occurred at a human’s mouth and throat regions to recognize the tasks. For instance, when a person speaks to someone, they generate vocal sounds, while eating and drinking produce chewing, sipping, and swallowing sounds. Several time- and frequency-domain features (e.g., zero-crossing rate and MFCCs) were used to train an SVM classifier.

Both algorithms achieved >70% accuracy during an in-the-wild study; therefore, the algorithms’ *sensitivity* is medium to high. Although the metrics used were reliable, the ensemble voting algorithm incorporated an environmentally embedded microphone, while the *BodyScope* sensor suffers from non-reproducibility; therefore, the algorithms’ *suitability* is classified as non-conforming. Finally, the algorithms’ *composite factor* and *concurrency* are classified as non-conforming.

#### 5.8.2 Deep learning

Deep learning algorithms can leverage the time, frequency and amplitude information in an audio signal’s spectrogram to extract the spatio-temporal features. Most auditory event detection deep learning algorithms (e.g., Laput et al., 2018; Haubrick and Ye, 2019; Liang and Thomaz, 2019) leverage transfer learning. These algorithms fine-tune the existing *VGGish model* (Hershey et al., 2017) [i.e., pre-trained on the *YouTube Audio Set* (Gemmeke et al., 2017)] with additional layers to detect the target auditory events. The *VGGish model* used a log Mel spectrogram as input to output a 128-dimensional feature embedding for every second of an audio sample. A transfer learning framework detected fifteen ADL tasks (e.g., talking, watching television, brushing, shaving, and listening to music) from the audio recorded using an off-the-shelf smartphone (Liang and Thomaz, 2019). A five-layer CNN was added to the *VGGish*’s feature embedding to predict the ADL tasks.

Polyphonic event detection algorithms recognize multiple auditory events occurring simultaneously (Chan and Chin, 2020), typically via deep neural networks. A *VGGish*-based algorithm stacked multiple binary classifiers to the feature vector (Haubrick and Ye, 2019), while others have developed various recurrent and hybrid deep neural networks to detect polyphonic sound events (Cakir et al., 2015; Parascandolo et al., 2016; Cakır et al., 2017).

Audio augmentation can be exploited by deep learning to improve the recognition rate. Augmenting the original audio with a set of deformations (e.g., time stretching, pitch shifting, dynamic range compression, and background noise mixing) improved a CNN’s classification accuracy significantly on a range of environmental sound classification tasks (Salamon and Bello, 2017). *Ubicoustics*, a real-time, auditory event recognition algorithm was trained by incorporating various augmentation techniques to simulate the sounds that resemble real-world audio samples (Laput et al., 2018).

Deep learning algorithms tend to outperform classical machine learning approaches in terms of accuracy. Thus, deep learning algorithms, especially the *VGGish*-based models (Laput et al., 2018; Haubrick and Ye, 2019; Liang and Thomaz, 2019), are the most suited for detecting auditory events, primarily due to their feature extraction capability and the availability of abundant audio datasets (Gemmeke et al., 2017; Mesaros et al., 2017). Most algorithms are validated by splitting all the available data randomly into training and validation datasets; therefore, the algorithms’ *generalizability* criteria either require additional evidence, or are non-conforming. All evaluated algorithms are non-conforming for the *composite factor* and *anomaly awareness* criteria.

#### 5.8.3 Discussion

Most auditory event detection algorithms typically have medium to high *sensitivity* (e.g., Stork et al., 2012; Yatani and Truong, 2012; Hershey et al., 2017; Laput et al., 2018; Haubrick and Ye, 2019). The algorithms’ *suitability* criterion depends on whether the microphone is worn or embedded in the environment. The algorithms’ *generalizability* criteria either require additional evidence or non-conforming. The polyphonic detection algorithms conform with concurrency; therefore, a deep polyphonic detection algorithm (e.g., Haubrick and Ye, 2019) is recommended for the intended HRT domain, as it is more likely to contain multiple, simultaneous sound sources (Chan and Chin, 2020). None of the reviewed algorithms conform with *composite factor* and *anomaly awareness*.

### 5.9 Speech tasks

Verbal communication plays a key role in task performance, such as assigning tasks, sharing or confirming important information, and reporting task completion, especially in a dynamic environment (Zhang and Sarcevic, 2015). Speech-reliant task recognition in a highly dynamic environment [e.g., trauma resuscitation (Bergs et al., 2005)] encounters several challenges, such as inconsistent verbal reports between tasks, the potentially succinct and non-grammatical nature of verbal communication, overlapping multi-person speech, and interleaved verbal exchanges due to multi-tasking (Jagannath et al., 2019). Only two speech task recognition algorithms were identified, their classifications are cited in Table 8.

**TABLE 8:** Speech task recognition algorithms’ evaluation overview.

Category	Paper	Sens	Suit	Genr	Comp	Conc	Anom
Algorithm							
Deep Learning							
Attention	Gu et al. (2019)	*∏*	NC	NC	NC	NC	NC
CNN	Abdulbaqi et al. (2020)	⋁	NC	NC	NC	NC	NC

#### 5.9.1 Deep learning

Little research exists on speech-reliant task recognition (Jagannath et al., 2018), with the existing algorithms being based on deep learning (e.g., Gu et al., 2019; Abdulbaqi et al., 2020). A text-based task recognition algorithm employed a verbal transcript derived from a medical team’s communication as input to predict ten trauma resuscitation tasks (Gu et al., 2019). The algorithm used both speech and ambient sounds for task prediction. A multimodal attention network was applied to process the transcribed spoken language and the ambient sounds in order to predict the tasks. The main limitation was reliance on manually-generated transcripts, which is infeasible for a contemporaneous task recognition system (Abdulbaqi, 2020). Automatic transcript generation requires a computationally expensive speech recognition tool. Additionally, poor audio quality caused by distant talking, ambient noise, succinct and non-grammatical speaking can increase an automatic speech recognition tool’s error rate; thus, the algorithm’s performance in real-world scenarios is expected to be lower than the cited result (Gu et al., 2019).

An alternative speech-reliant task recognition algorithm depended only on one keyword to detect trauma tasks (Abdulbaqi et al., 2020). The speech-reliant algorithm used one representative keyword per utterance as input to a deep neural network, in addition to the ambient sounds. This keyword was determined by calculating the most frequent words list for each task based on the premise that frequently occurring words for particular tasks can serve as features for the neural network’s task prediction. Word-spotting tools (e.g., Tsai and Hao, 2019; Gao et al., 2020) can extract keywords efficiently, reducing the reliance on traditional speech recognition. The deep learning architecture consisted of an audio network, a keyword network, and a fusion network. The audio network adapted a modified *VGGish* deep network (Simonyan and Zisserman, 2014) to extract features from the audio spectrogram, while the keyword network extracted important verbal features from the keyword list. The fusion network concatenated the output of both networks to predict the speech-reliant tasks.

The use of speech to recognize tasks is an under-developed area. The reviewed algorithms had low to medium *sensitivity* (e.g., Gu et al., 2019; Abdulbaqi et al., 2020). The algorithms’ *suitability* criterion was classified as non-conforming, because the incorporated metrics are domain and task specific. Both algorithms’ are non-conforming with *generalizability*, *composite factor*, *concurrency* and *anomaly awareness*.

#### 5.9.2 Discussion

Verbal communication in a highly dynamic setting [e.g., trauma medical team (Zhang and Sarcevic, 2015)] occurs at a high level (e.g., discussing task plans and intentions) and at a low level (e.g., coordinating, executing and reporting task completion) (Jagannath et al., 2018). Identifying speech patterns and keywords during verbal communication can detect the tasks, as well as track their progress (i.e., preparing-performing-reporting). Incorporating verbal exchanges [e.g., transcripts (Gu et al., 2019) or keywords (Abdulbaqi et al., 2020)] in tandem with the audio stream increased the accuracy by at least 15% in both algorithms, indicating that speech patterns and keywords can serve as differentiators for speech-reliant tasks. The algorithms’ main limitations are that the metrics are highly domain-specific and require natural language processing, along with substantial manual effort to identify task specific sensitive keywords (see Section 4). Therefore, the algorithms cannot be readily transferred across domains. A suitable alternative may involve modifying the algorithm to use the audio stream in conjunction with speech workload metrics (e.g., speech rate, voice intensity, and voice pitch) instead of verbal exchanges.

## 6 Discussion

HRTs often perform a wide range of tasks, such that the set of all tasks performed by human teammates may involve combinations of all activity components. Thus, an overall task recognition model capable of detecting tasks with multiple different activity components is hypothesized to improve the task recognition in more complex domains. None of the reviewed algorithms meet all the criteria necessary to achieve this goal, due to identifying tasks with a limited set of activity components, and the algorithms’ limitations with regard to the evaluation criteria: sensitivity, suitability, generalizability, composite factor, concurrency, and anomaly awareness.

Two algorithms, Grana et al.’s (2020) and Ishimaru et al.’s (2014a), come the closest to addressing the issues, as they identify four activity components. The former identified a gross motor task, while the latter identified a fine-grained motor task in addition to detecting visual, cognitive, and auditory tasks. However, both algorithms failed to satisfy all the required evaluation criteria. Most other algorithms detected tasks involving at most two activity components: gross and fine-grained motor, fine-grained motor and tactile, or visual and cognitive tasks. Finally, none of the reviewed algorithms detected tasks across all the identified activity components.

Several algorithms conform with sensitivity, suitability, and generalizability. Other than the cognitive and speech task recognition algorithms, there exists at least one algorithm that can detect tasks reliably while conforming with suitability and generalizability for each individual activity component. Existing algorithms are highly limited in satisfying the other three criteria: composite, concurrent, and anomaly awareness.

HRT tasks are typically composite in nature in that they are composed of differing combinations of multiple activity components; therefore, understanding the tasks’ various components is required in order to detect the tasks accurately, as well as track their progress. Thirteen algorithms, all of which were either gross or fine-grained motor task recognition algorithms, attempted to detect composite tasks. Eight algorithms detected composite tasks with high accuracy, while only three (Min and Cho, 2011; Liu et al., 2017; Liao et al., 2020) managed to detect tasks with high accuracy using reliable metrics from wearable sensors. A similar trend was observed for concurrency detection. Eight algorithms attempted to detect concurrent tasks, seven of which belonged to gross or fine-grained motor categories. Five of those algorithms detected tasks with high sensitivity, while only two (Liu et al., 2017; Liao et al., 2020) satisfied the sensitivity and suitability criteria.

Only two algorithms (Min et al., 2008; Laput and Harrison, 2019) conform with anomaly awareness. The remaining algorithms’ primary limitation for anomaly awareness was the assumption that humans will not perform tasks outside of a predefined set, which is not the case for the intended domain. Therefore, there remains a need for a task recognition algorithm that can detect out-of-class instances reliably. Such an algorithm can draw from existing novelty and anomaly detection research (e.g., Masana et al., 2018; Chalapathy and Chawla, 2019; Kwon et al., 2019; Perera and Patel, 2019; Pang et al., 2021).

## 7 Challenges and future directions

Despite the rapid growth in task recognition research, there are still a number of largely unexplored challenges. Some research challenges and future directions that can help realize the full potential of task recognition for uncertain, dynamic environments, especially within the context of first-response HRTs are presented.

• **Composite Task Recognition:** Existing task recognition methods can detect short-duration or repetitive atomic tasks (e.g., walking, running, reading) with high accuracy; however, detecting long-duration composite tasks (e.g., triaging a victim, digging a wildland fire trench) that involve multiple activity components remain difficult. A task recognition algorithm that incorporates several sensors as input to recognize the composite task’s multiple activity components is desired. For example, the algorithm can incorporate inertial and sEMG metrics to detect gross motor, fine-grained motor, and tactile tasks, as well as integrate pupil dilation, fixation, and saccades from a wearable eye tracker to detect visual tasks.
• **Leverage interactions between activity components:** Most composite tasks can be decomposed into atomic tasks across activity components. These atomic tasks typically follow a temporal ordering within the components. For example, *taking a picture of a suspicious object* requires *visually* scanning and *cognitively* evaluating the object prior to the *fine-grained motor* and *tactile* picture capturing task. A composite task recognition algorithm can leverage the interactions between the activity components in order to simultaneously (a) optimize each component’s atomic task detections, and (b) construct the composite tasks from the low-level atomic task detections.
• **Adaptively segmenting metrics:** Most existing task recognition algorithms segment the sensor data into temporal chunks using a fixed window size as input to the machine learning algorithms. A short-duration task may require a smaller window size, so that the task is not overshadowed (e.g., confused) by unrelated data, while a long-duration task may require a larger window size to have sufficient context. Therefore, it may be necessary for a task recognition algorithm to use an adaptive sliding window approach. This approach will permit expanding and contracting the window size based on the task, which may lead to more accurate detection. An ensemble learning algorithm may also be leveraged, where the algorithm makes predictions over multiple fixed window sizes and fuses the predictions across the window sizes intelligently to detect the tasks.
• **Lack of an ecologically-valid HRT dataset:** Machine learning algorithms only perform as well as the quality of the training data. Training and evaluating algorithms, especially deep learning models require large datasets. Most task recognition studies depend on existing benchmark datasets (e.g., *Opportunity* (Roggen et al., 2010), *WISDM* (Weiss et al., 2019), *PAMAP2* (Reiss and Stricker, 2012) that only involve day-to-day physical tasks and seldom incorporate composite tasks with multiple activity components. Additionally, most of these datasets only entail inertial and heart-rate metrics gathered in a highly controlled environment, which limits the algorithms’ capability to detect tasks across activity components. Datasets involving components other than the gross and fine-grained motor components are not available publicly. A composite, concurrent HRT task recognition evaluation that incorporates tasks involving multiple activity components performed in an ecologically valid setting is desired. A multimodal dataset that incorporates metrics acquired from multiple wearable sensors (e.g., whole-body inertial measurements, sEMG, eye tracking, and microphone audio) is required.

## 8 Conclusion

HRTs collaborating to achieve tasks under various conditions, especially in unstructured, dynamic environments, will require robots to adapt autonomously to a human teammate’s state. An important element of such adaptation is the robot’s ability to infer the human teammate’s current tasks, as understanding human actions and their interactions with the world provides the robot with more context as to what type of assistance the human may need. A multi-dimensional task recognition algorithm is needed to identify composite and atomic tasks performed by HRTs working in unstructured, dynamic environments in order to trigger such autonomous robot adaptations that can result in improved teaming outcomes.

This literature review classified task recognition algorithms based on six criteria: sensitivity, suitability, generalizability, composite factor, concurrency and anomaly awareness. The algorithms’ limitations include recognizing tasks with a limited set of activity component, not detecting composite and concurrent tasks across components, not being viable for HRT domain, and not accounting for individual differences.

## References

[B1] Abdoli-Eramaki M. Damecour C. Christenson J. Stevenson J. (2012). The effect of perspiration on the sEMG amplitude and power spectrum. J. Electromyogr. Kinesiol. 22, 908–913. 10.1016/j.jelekin.2012.04.009 22613823

[B2] Abdulbaqi J. Gu Y. Xu Z. Gao C. Marsic I. Burd R. S. (2020). Speech-based activity recognition for trauma resuscitation. IEEE Int. Conf. Healthc. Inf. 2020, 1–8. 10.1109/ichi48887.2020.9374372 PMC7962594 33738430

[B3] Abdulbaqi J. (2020). Speech-based activity recognition for medical teamwork. Ph.D. thesis. Rutgers University-School of Graduate Studies.

[B4] Ahlstrom U. Friedman-Berg F. J. (2006). Using eye movement activity as a correlate of cognitive workload. Int. J. Industrial Ergonomics 36, 623–636. 10.1016/j.ergon.2006.04.002

[B5] Akbari A. Jafari R. (2020). Personalizing activity recognition models through quantifying different types of uncertainty using wearable sensors. IEEE Trans. Biomed. Eng. 67, 2530–2541. 10.1109/tbme.2019.2963816 31905130

[B6] Al-qaness M. A. Dahou A. Abd Elaziz M. Helmi A. (2022). Multi-ResAtt: Multilevel residual network with attention for human activity recognition using wearable sensors. IEEE Trans. Industrial Inf. 19, 144–152. 10.1109/tii.2022.3165875

[B7] Allahbakhshi H. Conrow L. Naimi B. Weibel R. (2020). Using accelerometer and GPS data for real-life physical activity type detection. Sensors 20, 588. 10.3390/s20030588 31973129 PMC7038120

[B8] Allen J. F. Ferguson G. (1994). Actions and events in interval temporal logic. J. Log. Comput. 4, 531–579. 10.1093/logcom/4.5.531

[B9] Alsheikh M. A. Selim A. Niyato D. Doyle L. Lin S. Tan H.-P. (2016). “Deep activity recognition models with triaxial accelerometers,” in Workshops at the AAAI conference on artificial intelligence applied to assistive technologies and smart environments. WS-16-01.

[B10] Amma C. Krings T. Böer J. Schultz T. (2015). “Advancing muscle-computer interfaces with high-density electromyography,” in ACM conference on human factors in computing systems, 929–938.

[B11] Arif M. Bilal M. Kattan A. Ahamed S. I. (2014). Better physical activity classification using smartphone acceleration sensor. J. Med. Syst. 38, 95–10. 10.1007/s10916-014-0095-0 25000988

[B12] Atallah L. Lo B. King R. Yang G.-Z. (2010). “Sensor placement for activity detection using wearable accelerometers,” in IEEE international conference on body sensor networks, 24–29.

[B13] Atzori M. Gijsberts A. Castellini C. Caputo B. Hager A.-G. M. Elsig S. (2014). Electromyography data for non-invasive naturally-controlled robotic hand prostheses. Sci. Data 1, 140053–140113. 10.1038/sdata.2014.53 25977804 PMC4421935

[B14] Batzianoulis I. El-Khoury S. Pirondini E. Coscia M. Micera S. Billard A. (2017). EMG-based decoding of grasp gestures in reaching-to-grasping motions. Robotics Aut. Syst. 91, 59–70. 10.1016/j.robot.2016.12.014

[B15] Bergs E. A. Rutten F. L. Tadros T. Krijnen P. Schipper I. B. (2005). Communication during trauma resuscitation: Do we know what is happening? Injury 36, 905–911. 10.1016/j.injury.2004.12.047 15998511

[B16] Bian S. Liu M. Zhou B. Lukowicz P. (2022). The state-of-the-art sensing techniques in human activity recognition: A survey. Sensors 22, 4596. 10.3390/s22124596 35746376 PMC9229953

[B17] Biedert R. Hees J. Dengel A. Buscher G. (2012). “A robust realtime reading-skimming classifier,” in Eye tracking research and applications symposium, 123–130.

[B18] Bishop C. M. Nasrabadi N. M. (2006). Pattern recognition and machine learning, vol. 4. New York, NY: Springer.

[B19] Braojos R. Beretta I. Constantin J. Burg A. Atienza D. (2014). “A wireless body sensor network for activity monitoring with low transmission overhead,” in IEEE international conference on embedded and ubiquitous computing, 265–272.

[B20] Brendel W. Fern A. Todorovic S. (2011). “Probabilistic event logic for interval-based event recognition,” in IEEE international conference on computer vision and pattern recognition, 3329–3336.

[B21] Bulling A. Ward J. A. Gellersen H. Tröster G. (2010). Eye movement analysis for activity recognition using electrooculography. IEEE Trans. Pattern Analysis Mach. Intell. 33, 741–753. 10.1109/tpami.2010.86 20421675

[B22] Cakir E. Heittola T. Huttunen H. Virtanen T. (2015). “Polyphonic sound event detection using multi label deep neural networks,” in IEEE international joint conference on neural networks, 1–7.

[B23] Cakır E. Parascandolo G. Heittola T. Huttunen H. Virtanen T. (2017). Convolutional recurrent neural networks for polyphonic sound event detection. IEEE/ACM Trans. Audio, Speech, Lang. Process. 25, 1291–1303. 10.1109/taslp.2017.2690575

[B24] Capela N. A. Lemaire E. D. Baddour N. (2015). Feature selection for wearable smartphone-based human activity recognition with able bodied, elderly, and stroke patients. PLoS One 10, e0124414. 10.1371/journal.pone.0124414 25885272 PMC4401457

[B25] Castro D. Hickson S. Bettadapura V. Thomaz E. Abowd G. Christensen H. (2015). Predicting daily activities from egocentric images using deep learning. ACM Int. Symposium Wearable Comput. 2015, 75–82. 10.1145/2802083.2808398 PMC5851485 29553145

[B26] Cha S. H. Seo J. Baek S. H. Koo C. (2018). Towards a well-planned, activity-based work environment: Automated recognition of office activities using accelerometers. Build. Environ. 144, 86–93. 10.1016/j.buildenv.2018.07.051

[B27] Chalapathy R. Chawla S. (2019). Deep learning for anomaly detection: A survey. *arXiv preprint arXiv:1901.03407* .

[B28] Chan T. K. Chin C. S. (2020). A comprehensive review of polyphonic sound event detection. IEEE Access 8, 103339–103373. 10.1109/access.2020.2999388

[B29] Chen K. Zhang D. Yao L. Guo B. Yu Z. Liu Y. (2021a). Deep learning for sensor-based human activity recognition: Overview, challenges, and opportunities. ACM Comput. Surv. 54, 1–40. 10.1145/3447744

[B30] Chen L. Liu X. Peng L. Wu M. (2021b). Deep learning based multimodal complex human activity recognition using wearable devices. Appl. Intell. 51, 4029–4042. 10.1007/s10489-020-02005-7

[B31] Chen L. Nugent C. D. (2019). Human activity recognition and behaviour analysis. New York City, NY): Springer International Publishing.

[B32] Chen X. Zhang X. Zhao Z.-Y. Yang J.-H. Lantz V. Wang K.-Q. (2007a). “Hand gesture recognition research based on surface EMG sensors and 2D-accelerometers,” in IEEE international symposium on wearable computers, 11–14.

[B33] Chen X. Zhang X. Zhao Z.-Y. Yang J.-H. Lantz V. Wang K.-Q. (2007b). “Multiple hand gesture recognition based on surface EMG signal,” in IEEE international conference on bioinformatics and biomedical engineering, 506–509.

[B34] Chen Y. Xue Y. (2015). “A deep learning approach to human activity recognition based on single accelerometer,” in IEEE international conference on systems, man, and cybernetics, 1488–1492.

[B35] Chung P.-C. Liu C.-D. (2008). A daily behavior enabled hidden Markov model for human behavior understanding. Pattern Recognit. 41, 1572–1580. 10.1016/j.patcog.2007.10.022

[B36] Cleland I. Kikhia B. Nugent C. Boytsov A. Hallberg J. Synnes K. (2013). Optimal placement of accelerometers for the detection of everyday activities. Sensors 13, 9183–9200. 10.3390/s130709183 23867744 PMC3758644

[B37] Cornacchia M. Ozcan K. Zheng Y. Velipasalar S. (2017). A survey on activity detection and classification using wearable sensors. IEEE Sensors J. 17, 386–403. 10.1109/jsen.2016.2628346

[B38] Côté-Allard U. Fall C. L. Drouin A. Campeau-Lecours A. Gosselin C. Glette K. (2019). Deep learning for electromyographic hand gesture signal classification using transfer learning. IEEE Trans. Neural Syst. Rehabilitation Eng. 27, 760–771. 10.1109/tnsre.2019.2896269 30714928

[B39] Dash D. Ferrari P. Malik S. Wang J. (2019). “Automatic speech activity recognition from MEG signals using seq2seq learning,” in IEEE engineering in medicine and biology society international conference on neural engineering, 340–343.

[B40] Datta S. Banerjee A. Konar A. Tibarewala D. (2014). “Electrooculogram based cognitive context recognition,” in IEEE international conference on electronics, communication and instrumentation, 1–4.

[B41] Deng J. Dong W. Socher R. Li L.-J. Kai L. Li F.-F. (2009). “Imagenet: A large-scale hierarchical image database,” in IEEE conference on computer vision and pattern recognition, 248–255.

[B42] Ding H. Shangguan L. Yang Z. Han J. Zhou Z. Yang P. (2015). “Femo: A platform for free-weight exercise monitoring with RFIDs,” in ACM conference on embedded networked sensor systems, 141–154.

[B43] Dirgová Luptáková I. Kubovčík M. Pospíchal J. (2022). Wearable sensor-based human activity recognition with transformer model. Sensors 22, 1911. 10.3390/s22051911 35271058 PMC8914677

[B44] Du Y. Chen F. Xu W. Li Y. (2006). “Recognizing interaction activities using dynamic Bayesian network,” in IEEE international conference on pattern recognition 1, 618–621.

[B45] Elbasiony R. Gomaa W. (2020). “A survey on human activity recognition based on temporal signals of portable inertial sensors,” in International conference on advanced machine learning technologies and applications (Springer), 734–745.

[B46] EMOTIV (2021). Brainwear® - wireless EEG technology. Available at: http://emotiv.com/ (Accessed June 26, 2021).

[B47] Faisal A. I. Majumder S. Mondal T. Cowan D. Naseh S. Deen M. J. (2019). Monitoring methods of human body joints: State-of-the-art and research challenges. Sensors 19, 2629. 10.3390/s19112629 31185629 PMC6603670

[B48] Fan X. Wang F. Wang F. Gong W. Liu J. (2019). When RFID meets deep learning: Exploring cognitive intelligence for activity identification. IEEE Wirel. Commun. 26, 19–25. 10.1109/mwc.2019.1800405

[B49] Fathi A. Farhadi A. Rehg J. M. (2011). “Understanding egocentric activities,” in IEEE international conference on computer vision, 407–414.

[B50] Ferrari A. Micucci D. Mobilio M. Napoletano P. (2020). On the personalization of classification models for human activity recognition. IEEE Access 8, 32066–32079. 10.1109/access.2020.2973425

[B51] Fortin-Simard D. Bilodeau J.-S. Bouchard K. Gaboury S. Bouchard B. Bouzouane A. (2015). Exploiting passive RFID technology for activity recognition in smart homes. IEEE Intell. Syst. 30, 7–15. 10.1109/mis.2015.18

[B52] Fortune J. Heard J. Adams J. A. (2020). Real-time speech workload estimation for intelligent human-machine systems. Hum. Factors Ergonomics Soc. Annu. Meet. 64, 334–338. 10.1177/1071181320641076

[B53] Frank A. E. Kubota A. Riek L. D. (2019). “Wearable activity recognition for robust human-robot teaming in safety-critical environments via hybrid neural networks,” in IEEE/RSJ international conference on intelligent robots and systems, 449–454.

[B54] Gao Y. Mishchenko Y. Shah A. Matsoukas S. Vitaladevuni S. (2020). “Towards data-efficient modeling for wake word spotting,” in IEEE international conference on acoustics, speech and signal processing, 7479–7483.

[B55] Garcia-Hernando G. Yuan S. Baek S. Kim T.-K. (2018). “First-person hand action benchmark with RGB-D videos and 3d hand pose annotations,” in IEEE conference on computer vision and pattern recognition, 409–419.

[B56] Gemmeke J. F. Ellis D. P. Freedman D. Jansen A. Lawrence W. Moore R. C. (2017). “Audio set: An ontology and human-labeled dataset for audio events,” in IEEE international conference on acoustics, speech and signal processing, 776–780.

[B57] Gjoreski H. Bizjak J. Gjoreski M. Gams M. (2016). “Comparing deep and classical machine learning methods for human activity recognition using wrist accelerometer,” in International joint conference on artificial intelligence, workshop on deep learning for artificial intelligence, 10, 970.

[B58] Gjoreski H. Kozina S. Gams M. Luštrek M. (2014). “RAReFall—Real-time activity recognition and fall detection system,” in IEEE international conference on pervasive computing and communication workshops, 145–147.

[B59] Goodfellow I. Bengio Y. Courville A. (2016). Deep learning. Cambridge, MA, USA: MIT Press.

[B60] Grana M. Aguilar-Moreno M. De Lope Asiain J. Araquistain I. B. Garmendia X. (2020). Improved activity recognition combining inertial motion sensors and electroencephalogram signals. Int. J. Neural Syst. 30, 2050053. 10.1142/s0129065720500537 32917105

[B61] Granger C. W. (1969). Investigating causal relations by econometric models and cross-spectral methods. Econ. J. Econ. Soc. 37, 424–438. 10.2307/1912791

[B62] Granger C. W. (1980). Testing for causality: A personal viewpoint. J. Econ. Dyn. Control 2, 329–352. 10.1016/0165-1889(80)90069-x

[B63] Gu Y. Zhang R. Zhao X. Chen S. Abdulbaqi J. Marsic I. (2019). “Multimodal attention network for trauma activity recognition from spoken language and environmental sound,” in IEEE international conference on healthcare informatics, 1–6. 10.1109/ichi.2019.8904713 PMC7085888 32201857

[B64] Haubrick P. Ye J. (2019). “Robust audio sensing with multi-sound classification,” in IEEE international conference on pervasive computing and communications, 1–7.

[B65] He K. Zhang X. Ren S. Sun J. (2016). “Deep residual learning for image recognition,” in IEEE conference on computer vision and pattern recognition, 770–778.

[B66] Heard J. Fortune J. Adams J. A. (2019a). Speech workload estimation for human-machine interaction. Hum. Factors Ergonomics Soc. Annu. Meet. 63, 277–281. 10.1177/1071181319631018

[B67] Heard J. Harriott C. E. Adams J. A. (2018a). A survey of workload assessment algorithms. IEEE Trans. Human-Machine Syst. 48, 434–451. 10.1109/thms.2017.2782483

[B68] Heard J. Heald R. Harriott C. E. Adams J. A. (2018b). “A diagnostic human workload assessment algorithm for human-robot teams,” in ACM/IEEE international conference on human-robot interaction, 123–124.

[B69] Heard J. Paris R. A. Scully D. McNaughton C. Ehrenfeld J. M. Coco J. (2019b). Automatic clinical procedure detection for emergency services. IEEE Eng. Med. Biol. Soc. 2019, 337–340. 10.1109/EMBC.2019.8856281 31945910

[B70] Hershey S. Chaudhuri S. Ellis D. P. Gemmeke J. F. Jansen A. Moore R. C. (2017). “Cnn architectures for large-scale audio classification,” in IEEE international conference on acoustics, speech and signal processing, 131–135.

[B71] Hevesi P. Ward J. A. Amiraslanov O. Pirkl G. Lukowicz P. (2018). Wearable eye tracking for multisensor physical activity recognition. Int. J. Adv. Intelligent Syst. 10, 103–116.

[B72] Hsiao C.-P. Li R. Yan X. Do E. Y.-L. (2015). “Tactile teacher: Sensing finger tapping in piano playing,” in ACM international conference on tangible, embedded, and embodied interaction, 257–260.

[B73] Hu G. Qiu X. Meng L. (2016). “RTagCare: Deep human activity recognition powered by passive computational RFID sensors,” in IEEE asia-pacific network operations and management symposium, 1–4.

[B74] Ignatov A. (2018). Real-time human activity recognition from accelerometer data using convolutional neural networks. Appl. Soft Comput. 62, 915–922. 10.1016/j.asoc.2017.09.027

[B75] Inoue M. Inoue S. Nishida T. (2018). Deep recurrent neural network for mobile human activity recognition with high throughput. Artif. Life Robotics 23, 173–185. 10.1007/s10015-017-0422-x

[B76] Ishimaru S. Hoshika K. Kunze K. Kise K. Dengel A. (2017). “Towards reading trackers in the wild: Detecting reading activities by EOG glasses and deep neural networks,” in ACM international joint conference on pervasive and ubiquitous computing and ACM international symposium on wearable computers, 704–711.

[B77] Ishimaru S. Kunze K. Kise K. Weppner J. Dengel A. Lukowicz P. (2014a). “In the blink of an eye: Combining head motion and eye blink frequency for activity recognition with Google glass,” in ACM augmented human international conference, 1–4.

[B78] Ishimaru S. Kunze K. Uema Y. Kise K. Inami M. Tanaka K. (2014b). “Smarter eyewear: Using commercial EOG glasses for activity recognition,” in ACM international joint conference on pervasive and ubiquitous computing (Adjunct Publication), 239–242.

[B79] Islam M. R. Sakamoto S. Yamada Y. Vargo A. W. Iwata M. Iwamura M. (2021). Self-supervised learning for reading activity classification. Proc. ACM Interact. Mob. Wearable Ubiquitous Technol. 5, 1–22. 10.1145/3478088

[B80] Iwamoto T. Shinoda H. (2007). “Finger ring device for tactile sensing and human machine interface,” in Annual conference of society of instrument and control engineers, 2132–2136.

[B81] Jagannath S. Sarcevic A. Kamireddi N. Marsic I. (2019). “Assessing the feasibility of speech-based activity recognition in dynamic medical settings,” in ACM conference on human factors in computing systems, 1–6.

[B82] Jagannath S. Sarcevic A. Marsic I. (2018). “An analysis of speech as a modality for activity recognition during complex medical teamwork,” in ACM/EAI international conference on pervasive computing technologies for healthcare. 2018, 88–97. 10.1145/3240925.3240941 PMC6183054 30323960

[B83] Jalal A. Kamal S. Kim D. (2017). A depth video-based human detection and activity recognition using multi-features and embedded hidden Markov models for health care monitoring systems. Int. J. Interact. Multimedia Artif. Intell. 4, 54. 10.9781/ijimai.2017.447

[B84] Jia R. Liu B. (2013). “Human daily activity recognition by fusing accelerometer and multi-lead ECG data,” in IEEE international conference on signal processing, communication and computing, 1–4.

[B85] Jing L. Zhou Y. Cheng Z. Wang J. (2011). A recognition method for one-stroke finger gestures using a MEMS 3D accelerometer. IEICE Trans. Inf. Syst. 94, 1062–1072. 10.1587/transinf.e94.d.1062

[B86] Ju Z. Liu H. (2014). Human hand motion analysis with multisensory information. IEEE/ASME Trans. Mechatronics 19, 456–466. 10.1109/tmech.2013.2240312

[B87] Kaczmarek P. Mańkowski T. Tomczyński J. (2019). putEMG—a surface electromyography hand gesture recognition dataset. Sensors 19, 3548. 10.3390/s19163548 31416251 PMC6720505

[B88] Kawazoe A. Di Luca M. Visell Y. (2019). “Tactile echoes: A wearable system for tactile augmentation of objects,” in IEEE world haptics conference, 359–364.

[B89] Ke S.-R. Thuc H. L. U. Lee Y.-J. Hwang J.-N. Yoo J.-H. Choi K.-H. (2013). A review on video-based human activity recognition. Computers 2, 88–131. 10.3390/computers2020088

[B90] Kelton C. Wei Z. Ahn S. Balasubramanian A. Das S. R. Samaras D. (2019). “Reading detection in real-time,” in ACM symposium on eye tracking research and applications, 1–5.

[B91] Kher R. Pawar T. Thakar V. (2013). “Combining accelerometer data with Gabor energy feature vectors for body movements classification in ambulatory ECG signals,” in International conference on biomedical engineering and informatics, 413–417.

[B92] Kim Y.-J. Kang B.-N. Kim D. (2015). “Hidden Markov model ensemble for activity recognition using tri-axis accelerometer,” in IEEE international conference on systems, man, and cybernetics, 3036–3041.

[B93] Kolekar M. H. Dash D. P. (2016). “Hidden Markov model based human activity recognition using shape and optical flow based features,” in IEEE region 10 conference, 393–397.

[B94] Kollmorgen S. Holmqvist K. (2007). Automatically detecting reading in eye tracking data, 144. Lund University Cognitive Studies, 1–9.

[B95] Koskimaki H. Huikari V. Siirtola P. Laurinen P. Roning J. (2009). “Activity recognition using a wrist-worn inertial measurement unit: A case study for industrial assembly lines,” in IEEE mediterranean conference on control and automation, 401–405.

[B96] Koskimäki H. Siirtola P. Röning J. (2017). “MyoGym: Introducing an open gym data set for activity recognition collected using Myo armband,” in ACM international joint conference on pervasive and ubiquitous computing and ACM international symposium on wearable computers, 537–546.

[B97] Kubota A. Iqbal T. Shah J. A. Riek L. D. (2019). “Activity recognition in manufacturing: The roles of motion capture and sEMG+inertial wearables in detecting fine vs. gross motion,” in IEEE international conference on Robotics and automation, 6533–6539.

[B98] Kumar S. S. John M. (2016). “Human activity recognition using optical flow based feature set,” in IEEE international Carnahan conference on security technology, 1–5.

[B99] Kunze K. Kawaichi H. Yoshimura K. Kise K. (2013a). “Towards inferring language expertise using eye tracking,” in ACM extended abstracts on human factors in computing systems, 217–222.

[B100] Kunze K. Shiga Y. Ishimaru S. Kise K. (2013b). “Reading activity recognition using an off-the-shelf EEG – detecting reading activities and distinguishing genres of documents,” in IEEE international conference on document analysis and recognition, 96–100.

[B101] Kunze K. Utsumi Y. Shiga Y. Kise K. Bulling A. (2013c). “I know what you are reading: Recognition of document types using mobile eye tracking,” in ACM international symposium on wearable computers, 113–116.

[B102] Kwapisz J. R. Weiss G. M. Moore S. A. (2011). Activity recognition using cell phone accelerometers. ACM SigKDD Explor. Newsl. 12, 74–82. 10.1145/1964897.1964918

[B103] Kwon D. Kim H. Kim J. Suh S. C. Kim I. Kim K. J. (2019). A survey of deep learning-based network anomaly detection. Clust. Comput. 22, 949–961. 10.1007/s10586-017-1117-8

[B104] Ladjailia A. Bouchrika I. Merouani H. F. Harrati N. Mahfouf Z. (2020). Human activity recognition via optical flow: Decomposing activities into basic actions. Neural Comput. Appl. 32, 16387–16400. 10.1007/s00521-018-3951-x

[B105] Lagodzinski P. Shirahama K. Grzegorzek M. (2018). Codebook-based electrooculography data analysis towards cognitive activity recognition. Comput. Biol. Med. 95, 277–287. 10.1016/j.compbiomed.2017.10.026 29126580

[B106] Lan G. Heit B. Scargill T. Gorlatova M. (2020). “GazeGraph: Graph-based few-shot cognitive context sensing from human visual behavior,” in Embedded networked sensor systems, 422–435.

[B107] Landsmann M. Augereau O. Kise K. (2019). “Classification of reading and not reading behavior based on eye movement analysis,” in ACM international joint conference on pervasive and ubiquitous computing and ACM international symposium on wearable computers, 109–112.

[B108] Laput G. Ahuja K. Goel M. Harrison C. (2018). “Ubicoustics: Plug-and-play acoustic activity recognition,” in ACM symposium on user interface software and technology, 213–224.

[B109] Laput G. Harrison C. (2019). “Sensing fine-grained hand activity with smartwatches,” in ACM conference on human factors in computing systems.

[B110] Laput G. Yang C. Xiao R. Sample A. Harrison C. (2015). “EM-Sense: Touch recognition of uninstrumented, electrical and electromechanical objects,” in ACM symposium on user interface software and technology (New York, NY, USA: Association for Computing Machinery), 157–166.

[B111] Lara O. D. Labrador M. A. (2013). A survey on human activity recognition using wearable sensors. IEEE Commun. Surv. Tutorials 15, 1192–1209. 10.1109/surv.2012.110112.00192

[B112] Lara O. D. Pérez A. J. Labrador M. A. Posada J. D. (2012). Centinela: A human activity recognition system based on acceleration and vital sign data. Pervasive Mob. Comput. 8, 717–729. 10.1016/j.pmcj.2011.06.004

[B113] Lee K.-F. Hon H.-W. Reddy R. (1990). An overview of the SPHINX speech recognition system. IEEE Trans. Acoust. Speech, Signal Process. 38, 35–45. 10.1109/29.45616

[B114] Lee M. H. Nicholls H. R. (1999). Review article tactile sensing for mechatronics—A state of the art survey. Mechatronics 9, 1–31. 10.1016/s0957-4158(98)00045-2

[B115] Lee S.-M. Yoon S. M. Cho H. (2017). “Human activity recognition from accelerometer data using convolutional neural network,” in IEEE international conference on big data and smart computing, 131–134.

[B116] Lee Y.-S. Cho S.-B. (2011). “Activity recognition using hierarchical hidden Markov models on a smartphone with 3d accelerometer,” in International conference on hybrid artificial intelligence systems (Springer), 460–467.

[B117] Li L. Paris R. A. Pinson C. Wang Y. Coco J. Heard J. (2020a). Emergency clinical procedure detection with deep learning. IEEE Eng. Med. Biol. Soc. 2020, 158–163. 10.1109/EMBC44109.2020.9175575 33017954

[B118] Li M. Rozgić V. Thatte G. Lee S. Emken A. Annavaram M. (2010). Multimodal physical activity recognition by fusing temporal and cepstral information. IEEE Trans. Neural Syst. Rehabilitation Eng. 18, 369–380. 10.1109/tnsre.2010.2053217 PMC4326092 20699202

[B119] Li X. Luo J. Younes R. (2020b). “Activitygan: Generative adversarial networks for data augmentation in sensor-based human activity recognition,” in ACM international joint conference on pervasive and ubiquitous computing, 249–254.

[B120] Li X. Yao D. Pan X. Johannaman J. Yang J. Webman R. (2016a). “Activity recognition for medical teamwork based on passive RFID,” in IEEE international conference on RFID, 1–9. 10.1109/RFID.2016.7488002 PMC6200354 30370332

[B121] Li X. Zhang Y. Marsic I. Sarcevic A. Burd R. S. (2016b). Deep learning for RFID-based activity recognition. ACM Conf. Embed. Netw. Sens. Syst. 2016, 164–175. 10.1145/2994551.2994569 PMC6205502 30381808

[B122] Liang D. Thomaz E. (2019). Audio-based activities of daily living (ADL) recognition with large-scale acoustic embeddings from online videos. ACM Interact. Mob. Wearable Ubiquitous Technol. 3, 1–18. 10.1145/3314404

[B123] Liao J. Hu J. Liu L. (2020). “Recognizing complex activities by a temporal causal network-based model,” in Joint European conference on machine learning and knowledge discovery in databases (Springer), 341–357.

[B124] Liao L. Fox D. Kautz H. (2007). “Hierarchical conditional random fields for GPS-based activity recognition,” in Robotics research (Springer), 487–506.

[B125] Lillo I. Niebles J. C. Soto A. (2017). Sparse composition of body poses and atomic actions for human activity recognition in RGB-D videos. Image Vis. Comput. 59, 63–75. 10.1016/j.imavis.2016.11.004

[B126] Liu J. Liu H. Chen Y. Wang Y. Wang C. (2019). Wireless sensing for human activity: A survey. IEEE Commun. Surv. Tutorials 22, 1629–1645. 10.1109/comst.2019.2934489

[B127] Liu L. Wang S. Su G. Huang Z.-G. Liu M. (2017). Towards complex activity recognition using a Bayesian network-based probabilistic generative framework. Pattern Recognit. 68, 295–309. 10.1016/j.patcog.2017.02.028

[B128] Liu X. Rajan S. Ramasarma N. Bonato P. Lee S. I. (2018). The use of a finger-worn accelerometer for monitoring of hand use in ambulatory settings. IEEE J. Biomed. Health Inf. 23, 599–606. 10.1109/jbhi.2018.2821136 29994103

[B129] Lu Y. Zhang C. Zhou B.-Y. Gao X.-P. Lv Z. (2018). A dual model approach to EOG-based human activity recognition. Biomed. Signal Process. Control 45, 50–57. 10.1016/j.bspc.2018.05.011

[B130] Ma M. Fan H. Kitani K. M. (2016). “Going deeper into first-person activity recognition,” in IEEE conference on computer vision and pattern recognition, 1894–1903.

[B131] Mannini A. Intille S. S. (2018). Classifier personalization for activity recognition using wrist accelerometers. IEEE J. Biomed. health Inf. 23, 1585–1594. 10.1109/jbhi.2018.2869779 PMC6639791 30222588

[B132] Mannini A. Sabatini A. M. (2011). On-line classification of human activity and estimation of walk-run speed from acceleration data using support vector machines. IEEE Eng. Med. Biol. Soc. 2011, 3302–3305. 10.1109/IEMBS.2011.6090896 22255045

[B133] Marquart G. Cabrall C. de Winter J. (2015). Review of eye-related measures of drivers’ mental workload. Procedia Manuf. 3, 2854–2861. 10.1016/j.promfg.2015.07.783

[B134] Martinez F. Pissaloux E. Carbone A. (2017). Towards activity recognition from eye-movements using contextual temporal learning. Integr. Computer-Aided Eng. 24, 1–16. 10.3233/ica-160520

[B135] Masana M. Ruiz I. Serrat J. van de Weijer J. Lopez A. M. (2018). Metric learning for novelty and anomaly detection. *arXiv preprint arXiv:1808.05492* .

[B136] Matsuo K. Yamada K. Ueno S. Naito S. (2014). “An attention-based activity recognition for egocentric video,” in IEEE conference on computer vision and pattern recognition workshops, 565–570.

[B137] Mayol W. W. Murray D. W. (2005). “Wearable hand activity recognition for event summarization,” in IEEE international symposium on wearable computers, 122–129.

[B138] Mekruksavanich S. Jitpattanakul A. Sitthithakerngkiet K. Youplao P. Yupapin P. (2022). Resnet-se: Channel attention-based deep residual network for complex activity recognition using wrist-worn wearable sensors. IEEE Access 10, 51142–51154. 10.1109/access.2022.3174124

[B139] Mesaros A. Heittola T. Diment A. Elizalde B. Shah A. Vincent E. (2017). “DCASE 2017 challenge setup: Tasks, datasets and baseline system,” in Detection and classification of acoustic scenes and events workshops.

[B140] Meyer J. Frank A. Schlebusch T. Kasneci E. (2022). U-HAR: A convolutional approach to human activity recognition combining head and eye movements for context-aware smart glasses. ACM Human-Computer Interact. 6, 1–19. 10.1145/3530884

[B141] Min C.-H. Ince N. F. Tewfik A. H. (2008). “Early morning activity detection using acoustics and wearable wireless sensors,” in IEEE European signal processing conference, 1–5. 10.1109/IEMBS.2008.4650384 19163887

[B142] Min C. Ince N. F. Tewfik A. H. (2007). “Generalization capability of a wearable early morning activity detection system,” in IEEE European signal processing conference, 1556–1560.

[B143] Min J. Cho S. (2011). “Activity recognition based on wearable sensors using selection/fusion hybrid ensemble,” in IEEE international conference on systems, man, and cybernetics, 1319–1324.

[B144] Mostow J. Chang K.-M. Nelson J. (2011). “Toward exploiting EEG input in a reading tutor,” in International conference on artificial intelligence in education (Springer), 230–237.

[B145] MUSE (2021). Interaxon inc., MuseTM. Available at: https://choosemuse.com/muse-2/ (Accessed June 26, 2021).

[B146] Nandy A. Saha J. Chowdhury C. (2020). Novel features for intensive human activity recognition based on wearable and smartphone sensors. Microsyst. Technol. 26, 1889–1903. 10.1007/s00542-019-04738-z

[B147] Neili Boualia S. Essoukri Ben Amara N. (2021). “Deep full-body HPE for activity recognition from RGB frames only,” in Informatics (MDPI), 8, 2. 10.3390/informatics8010002

[B148] Neurosky (2021). Neurosky, biosensors, Neurosky co. Available at: http://neurosky.com/ (Accessed June 26, 2021).

[B149] Nweke H. F. Teh Y. W. Al-Garadi M. A. Alo U. R. (2018). Deep learning algorithms for human activity recognition using mobile and wearable sensor networks: State of the art and research challenges. Expert Syst. Appl. 105, 233–261. 10.1016/j.eswa.2018.03.056

[B150] Ordóñez F. J. Roggen D. (2016). Deep convolutional and lstm recurrent neural networks for multimodal wearable activity recognition. Sensors 16, 115. 10.3390/s16010115 26797612 PMC4732148

[B151] Orr C. Nugent C. Wang H. Zheng H. (2018). “A multi agent approach to facilitate the identification of interleaved activities,” in International conference on digital health, 126–130.

[B152] Ozioko O. Taube W. Hersh M. Dahiya R. (2017). “SmartFingerBraille: A tactile sensing and actuation based communication glove for deafblind people,” in IEEE international symposium on industrial electronics, 2014–2018.

[B153] Pang G. Shen C. Cao L. Hengel A. V. D. (2021). Deep learning for anomaly detection: A review. ACM Comput. Surv. 54, 1–38. 10.1145/3439950

[B154] Parascandolo G. Huttunen H. Virtanen T. (2016). “Recurrent neural networks for polyphonic sound event detection in real life recordings,” in IEEE international conference on acoustics, speech and signal processing, 6440–6444.

[B155] Pareek P. Thakkar A. (2021). A survey on video-based human action recognition: Recent updates, datasets, challenges, and applications. Artif. Intell. Rev. 54, 2259–2322. 10.1007/s10462-020-09904-8

[B156] Park H. Dong S.-Y. Lee M. Youn I. (2017). The role of heart-rate variability parameters in activity recognition and energy-expenditure estimation using wearable sensors. Sensors 17, 1698. 10.3390/s17071698 28737732 PMC5539477

[B157] Parkka J. Ermes M. Korpipaa P. Mantyjarvi J. Peltola J. Korhonen I. (2006). Activity classification using realistic data from wearable sensors. IEEE Trans. Inf. Technol. Biomed. 10, 119–128. 10.1109/titb.2005.856863 16445257

[B158] Pawar T. Anantakrishnan N. Chaudhuri S. Duttagupta S. P. (2007). Transition detection in body movement activities for wearable ECG. IEEE Trans. Biomed. Eng. 54, 1149–1152. 10.1109/tbme.2007.891950 17549906

[B159] Pawar T. Chaudhuri S. Duttagupta S. P. (2006). “Analysis of ambulatory ECG signal,” in IEEE engineering in medicine and biology society, 3094–3097. 10.1109/IEMBS.2006.259315 17945754

[B160] Peng L. Chen L. Ye Z. Zhang Y. (2018). Aroma: A deep multi-task learning based simple and complex human activity recognition method using wearable sensors. ACM Interact. Mob. Wearable Ubiquitous Technol. 2, 1–16. 10.1145/3214277

[B161] Pennington J. Socher R. Manning C. D. (2014). “Glove: Global vectors for word representation,” in Conference on empirical methods in natural language processing, 1532–1543.

[B162] Perera P. Patel V. M. (2019). “Deep transfer learning for multiple class novelty detection,” in IEEE/CVF conference on computer vision and pattern recognition, 11544–11552.

[B163] Pirsiavash H. Ramanan D. (2012). “Detecting activities of daily living in first-person camera views,” in IEEE conference on computer vision and pattern recognition, 2847–2854.

[B164] Porter G. Troscianko T. Gilchrist I. D. (2007). Effort during visual search and counting: Insights from pupillometry. Q. J. Exp. Psychol. 60, 211–229. 10.1080/17470210600673818 17455055

[B165] Povey D. Ghoshal A. Boulianne G. Burget L. Glembek O. Goel N. (2011). “The Kaldi speech recognition toolkit,” in IEEE workshop on automatic speech recognition and understanding (IEEE Signal Processing Society), CFP11SRW-USB).

[B166] Pratap V. Hannun A. Xu Q. Cai J. Kahn J. Synnaeve G. (2019). “Wav2letter++: A fast open-source speech recognition system,” in IEEE international conference on acoustics, speech and signal processing, 6460–6464.

[B167] Privitera C. M. Renninger L. W. Carney T. Klein S. Aguilar M. (2010). Pupil dilation during visual target detection. J. Vis. 10, 3–14. 10.1167/10.10.3 20884468

[B168] Rahimian E. Zabihi S. Asif A. Mohammadi A. (2020). “Hybrid deep neural networks for sparse surface EMG-based hand gesture recognition,” in IEEE asilomar conference on signals, systems, and computers, 371–374.

[B169] Ramanujam E. Perumal T. Padmavathi S. (2021). Human activity recognition with smartphone and wearable sensors using deep learning techniques: A review. IEEE Sensors J. 21, 13029–13040. 10.1109/jsen.2021.3069927

[B170] Ravi N. Dandekar N. Mysore P. Littman M. L. (2005). “Activity recognition from accelerometer data,” in AAAI conference on innovative applications of artificial intelligence, 1541–1546.

[B171] Reddy S. Mun M. Burke J. Estrin D. Hansen M. Srivastava M. (2010). Using mobile phones to determine transportation modes. ACM Trans. Sens. Netw. 6, 1–27. 10.1145/1689239.1689243

[B172] Redmon J. Divvala S. Girshick R. Farhadi A. (2016). “You only look once: Unified, real-time object detection,” in IEEE conference on computer vision and pattern recognition, 779–788.

[B173] Reiss A. Stricker D. (2012). “Introducing a new benchmarked dataset for activity monitoring,” in International symposium on wearable computers (IEEE), 108–109.

[B174] Riboni D. Bettini C. (2011). Cosar: Hybrid reasoning for context-aware activity recognition. Personal Ubiquitous Comput. 15, 271–289. 10.1007/s00779-010-0331-7

[B175] Roggen D. Calatroni A. Rossi M. Holleczek T. Förster K. Tröster G. (2010). “Collecting complex activity datasets in highly rich networked sensor environments,” in IEEE international conference on networked sensing systems, 233–240.

[B176] Saez Y. Baldominos A. Isasi P. (2017). A comparison study of classifier algorithms for cross-person physical activity recognition. Sensors 17, 66. 10.3390/s17010066 PMC5298639 28042838

[B177] Safyan M. Qayyum Z. U. Sarwar S. García-Castro R. Ahmed M. (2019). Ontology-driven semantic unified modelling for concurrent activity recognition (OSCAR). Multimedia Tools Appl. 78, 2073–2104. 10.1007/s11042-018-6318-5

[B178] Saguna S. Zaslavsky A. Chakraborty D. (2013). Complex activity recognition using context-driven activity theory and activity signatures. ACM Trans. Computer-Human Interact. 20, 1–34. 10.1145/2490832

[B179] Salamon J. Bello J. P. (2017). Deep convolutional neural networks and data augmentation for environmental sound classification. IEEE Signal Process. Lett. 24, 279–283. 10.1109/lsp.2017.2657381

[B180] Salehzadeh A. Calitz A. P. Greyling J. (2020). Human activity recognition using deep electroencephalography learning. Biomed. Signal Process. Control 62, 102094. 10.1016/j.bspc.2020.102094

[B181] Salvador S. Chan P. (2007). Toward accurate dynamic time warping in linear time and space. Intell. Data Anal. 11, 561–580. 10.3233/ida-2007-11508

[B182] Sarkar S. Reddy K. Dorgan A. Fidopiastis C. Giering M. (2016). Wearable EEG-based activity recognition in phm-related service environment via deep learning. Int. J. Prognostics Health Manag. 7, 1–10. 10.36001/ijphm.2016.v7i4.2459

[B183] Sathiyanarayanan M. Rajan S. (2016). “Myo armband for physiotherapy healthcare: A case study using gesture recognition application,” in IEEE international conference on communication systems and networks, 1–6.

[B184] Scheme E. Englehart K. (2011). Electromyogram pattern recognition for control of powered upper-limb prostheses: State of the art and challenges for clinical use. J. Rehabilitation Res. Dev. 48, 643–659. 10.1682/jrrd.2010.09.0177 21938652

[B185] Scherhäufl M. Pichler M. Stelzer A. (2014). UHF RFID localization based on phase evaluation of passive tag arrays. IEEE Trans. Instrum. Meas. 64, 913–922. 10.1109/tim.2014.2363578

[B186] Schirrmeister R. T. Springenberg J. T. Fiederer L. D. J. Glasstetter M. Eggensperger K. Tangermann M. (2017). Deep learning with convolutional neural networks for EEG decoding and visualization. Hum. Brain Mapp. 38, 5391–5420. 10.1002/hbm.23730 28782865 PMC5655781

[B187] Schuldt C. Laptev I. Caputo B. (2004). “Recognizing human actions: A local SVM approach,” in IEEE international conference on pattern recognition, 3, 32–36.

[B188] Sha X. Lian C. Zhao Y. Yu J. Li W. J. (2020). An explicable keystroke recognition algorithm for customizable ring-type keyboards. IEEE Access 8, 22933–22944. 10.1109/access.2020.2968495

[B189] Shakya S. R. Zhang C. Zhou Z. (2018). Comparative study of machine learning and deep learning architecture for human activity recognition using accelerometer data. Int. J. Mach. Learn. Comput. 8, 577–582.

[B190] Simonyan K. Zisserman A. (2014). Very deep convolutional networks for large-scale image recognition. *arXiv preprint arXiv:1409.1556* .

[B191] Srivastava N. Newn J. Velloso E. (2018). Combining low and mid-level gaze features for desktop activity recognition. ACM Interact. Mob. Wearable Ubiquitous Technol. 2, 1–27. 10.1145/3287067

[B192] Steil J. Bulling A. (2015). “Discovery of everyday human activities from long-term visual behaviour using topic models,” in ACM international joint conference on pervasive and ubiquitous computing, 75–85.

[B193] Stork J. A. Spinello L. Silva J. Arras K. O. (2012). “Audio-based human activity recognition using non-markovian ensemble voting,” in IEEE international symposium on robot and human interactive communication, 509–514.

[B194] Tang Y. Lu J. Wang Z. Yang M. Zhou J. (2019). Learning semantics-preserving attention and contextual interaction for group activity recognition. IEEE Trans. Image Process. 28, 4997–5012. 10.1109/tip.2019.2914577 31071041

[B195] Tapia E. M. Intille S. S. Haskell W. Larson K. Wright J. King A. (2007). “Real-time recognition of physical activities and their intensities using wireless accelerometers and a heart rate monitor,” in IEEE international symposium on wearable computers, 37–40.

[B196] Thu N. T. H. Han D. S. (2021). Hihar: A hierarchical hybrid deep learning architecture for wearable sensor-based human activity recognition. IEEE Access 9, 145271–145281. 10.1109/access.2021.3122298

[B197] Triboan D. Chen L. Chen F. Wang Z. (2017). Semantic segmentation of real-time sensor data stream for complex activity recognition. Personal Ubiquitous Comput. 21, 411–425. 10.1007/s00779-017-1005-5

[B198] Trigili E. Grazi L. Crea S. Accogli A. Carpaneto J. Micera S. (2019). Detection of movement onset using EMG signals for upper-limb exoskeletons in reaching tasks. J. Neuroengineering Rehabilitation 16, 45–16. 10.1186/s12984-019-0512-1 PMC6440169 30922326

[B199] Tsai T.-H. Hao P.-C. (2019). “Customized wake-up word with key word spotting using convolutional neural network,” in IEEE international SoC design conference, 136–137.

[B200] Ullah A. Muhammad K. Del Ser J. Baik S. W. de Albuquerque V. H. C. (2018). Activity recognition using temporal optical flow convolutional features and multilayer lstm. IEEE Trans. Industrial Electron. 66, 9692–9702. 10.1109/tie.2018.2881943

[B201] Vail D. L. Veloso M. M. Lafferty J. D. (2007). “Conditional random fields for activity recognition,” in International joint conference on autonomous agents and multiagent systems, 1–8.

[B202] Vepakomma P. De D. Das S. K. Bhansali S. (2015). “A-Wristocracy: Deep learning on wrist-worn sensing for recognition of user complex activities,” in IEEE international conference on wearable and implantable body sensor networks, 1–6.

[B203] Wahn B. Ferris D. P. Hairston W. D. König P. (2016). Pupil sizes scale with attentional load and task experience in a multiple object tracking task. PLOS ONE 11, 01680877–e168115. 10.1371/journal.pone.0168087 PMC5157994 27977762

[B204] Wang E. J. Lee T.-J. Mariakakis A. Goel M. Gupta S. Patel S. N. (2015). “Magnifisense: Inferring device interaction using wrist-worn passive magneto-inductive sensors,” in ACM international joint conference on pervasive and ubiquitous computing, 15–26.

[B205] Wang J. Chen Y. Gu Y. Xiao Y. Pan H. (2018). “Sensorygans: An effective generative adversarial framework for sensor-based human activity recognition,” in International joint conference on neural networks (IEEE), 1–8.

[B206] Wang J. Chen Y. Hao S. Peng X. Hu L. (2019). Deep learning for sensor-based activity recognition: A survey. Pattern Recognit. Lett. 119, 3–11. 10.1016/j.patrec.2018.02.010

[B207] Wang Z. Jiang M. Hu Y. Li H. (2012). An incremental learning method based on probabilistic neural networks and adjustable fuzzy clustering for human activity recognition by using wearable sensors. IEEE Trans. Inf. Technol. Biomed. 16, 691–699. 10.1109/titb.2012.2196440 22614724

[B208] Weiss G. M. Yoneda K. Hayajneh T. (2019). Smartphone and smartwatch-based biometrics using activities of daily living. IEEE Access 7, 133190–133202. 10.1109/access.2019.2940729

[B209] Weng S. Xiang L. Tang W. Yang H. Zheng L. Lu H. (2014). “A low power and high accuracy mems sensor based activity recognition algorithm,” in IEEE international conference on bioinformatics and biomedicine, 33–38.

[B210] Wu D. Sharma N. Blumenstein M. (2017). “Recent advances in video-based human action recognition using deep learning: A review,” in International joint conference on neural networks (IEEE), 2865–2872.

[B211] Wu J. Osuntogun A. Choudhury T. Philipose M. Rehg J. M. (2007). “A scalable approach to activity recognition based on object use,” in IEEE international conference on computer vision, 1–8.

[B212] Xu C. Chai D. He J. Zhang X. Duan S. (2019). Innohar: A deep neural network for complex human activity recognition. IEEE Access 7, 9893–9902. 10.1109/access.2018.2890675

[B213] Yatani K. Truong K. N. (2012). “Bodyscope: A wearable acoustic sensor for activity recognition,” in ACM conference on ubiquitous computing, 341–350.

[B214] Yuan Y. Chang K.-M. Taylor J. N. Mostow J. (2014). “Toward unobtrusive measurement of reading comprehension using low-cost EEG,” in International conference on learning analytics and knowledge, 54–58.

[B215] Zhang H.-B. Zhang Y.-X. Zhong B. Lei Q. Yang L. Du J.-X. (2019a). A comprehensive survey of vision-based human action recognition methods. Sensors 19, 1005. 10.3390/s19051005 30818796 PMC6427144

[B216] Zhang S. Ang M. Xiao W. Tham C. (2008). “Detection of activities for daily life surveillance: Eating and drinking,” in IEEE international conference on eHealth networking, applications and services, 171–176.

[B217] Zhang S. Li Y. Zhang S. Shahabi F. Xia S. Deng Y. (2022a). Deep learning in human activity recognition with wearable sensors: A review on advances. Sensors 22, 1476. 10.3390/s22041476 35214377 PMC8879042

[B218] Zhang X. Chen X. Li Y. Lantz V. Wang K. Yang J. (2011). A framework for hand gesture recognition based on accelerometer and EMG sensors. IEEE Trans. Syst. Man, Cybernetics-Part A Syst. Humans 41, 1064–1076. 10.1109/tsmca.2011.2116004

[B219] Zhang X. Chen X. Wang W.-H. Yang J.-H. Lantz V. Wang K.-Q. (2009). “Hand gesture recognition and virtual game control based on 3D accelerometer and EMG sensors,” in ACM international conference on intelligent user interfaces, 401–406.

[B220] Zhang X. Yao L. Wang X. Zhang W. Zhang S. Liu Y. (2019b). “Know your mind: Adaptive cognitive activity recognition with reinforced cnn,” in IEEE international conference on data mining, 896–905.

[B221] Zhang Y. Harrison C. (2015). “Tomo: Wearable, low-cost electrical impedance tomography for hand gesture recognition,” in ACM symposium on user interface software and technology, 167–173.

[B222] Zhang Y. Wang L. Chen H. Tian A. Zhou S. Guo Y. (2022b). IF-ConvTransformer: A framework for human activity recognition using imu fusion and convtransformer. ACM Interact. Mob. Wearable Ubiquitous Technol. 6, 1–26. 10.1145/3534584

[B223] Zhang Y. Zhang Y. Swears E. Larios N. Wang Z. Ji Q. (2013). Modeling temporal interactions with interval temporal Bayesian networks for complex activity recognition. IEEE Trans. Pattern Analysis Mach. Intell. 35, 2468–2483. 10.1109/tpami.2013.33 23969390

[B224] Zhang Z. Sarcevic A. (2015). “Constructing awareness through speech, gesture, gaze and movement during a time-critical medical task,” in European conference on computer supported cooperative work (Springer), 163–182.

[B225] Zhao Y. Yang R. Chevalier G. Xu X. Zhang Z. (2018). Deep residual bidir-LSTM for human activity recognition using wearable sensors. Math. Problems Eng. 2018, 1–13. 10.1155/2018/7316954

[B226] Zhou Y. Cheng Z. Jing L. (2015). Threshold selection and adjustment for online segmentation of one-stroke finger gestures using single tri-axial accelerometer. Multimedia Tools Appl. 74, 9387–9406. 10.1007/s11042-014-2111-2

